# Supplementary figures and images for: Comprehensive analysis of peroxisome proliferator-activated receptors to predict the drug resistance, immune microenvironment, and prognosis in stomach adenocarcinomas
Source: PeerJ. 2024 Mar 22;12:e17082. doi: 10.7717/peerj.17082 (PMC10962337; doi:10.7717/peerj.17082)

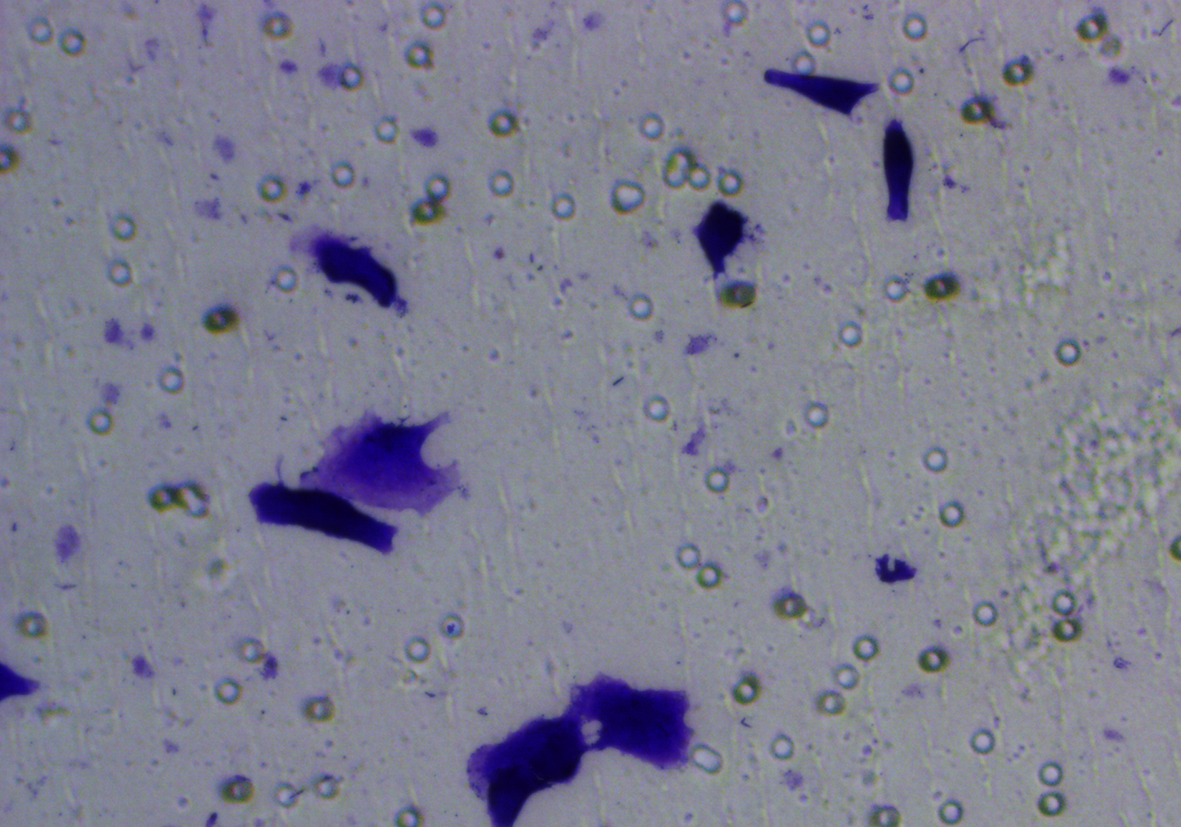

Supplement: Supplemental Information 1 [file peerj-12-17082-s001.zip › AGS-SI-I/AGS-SI-I (1).jpg]

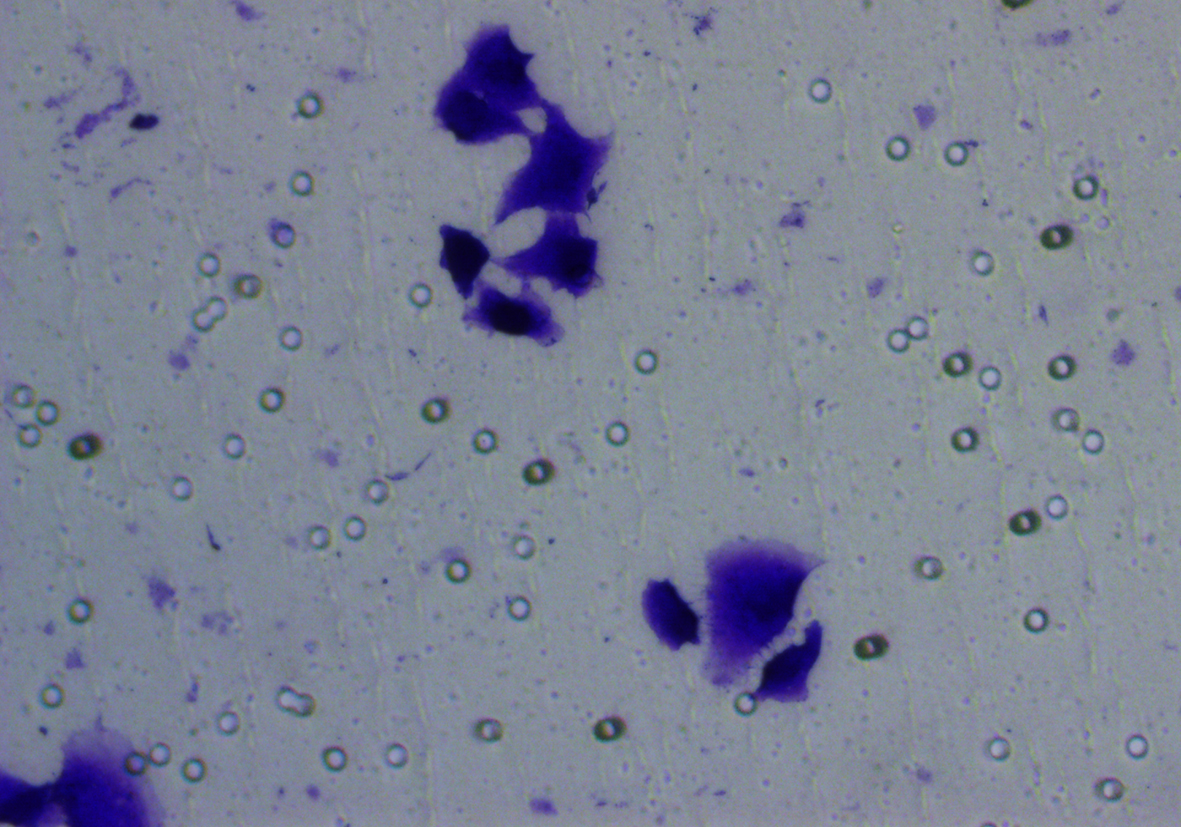

Supplement: Supplemental Information 1 [file peerj-12-17082-s001.zip › AGS-SI-I/AGS-SI-I (2).jpg]

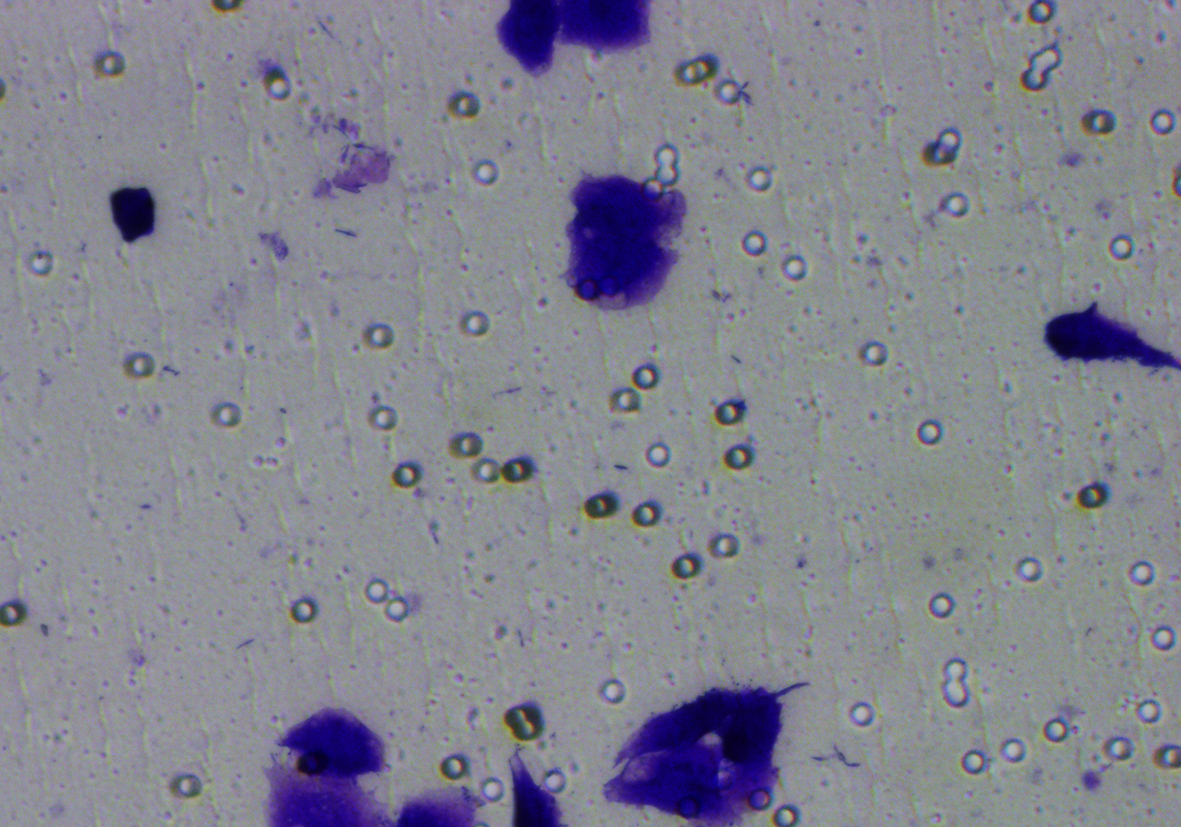

Supplement: Supplemental Information 1 [file peerj-12-17082-s001.zip › AGS-SI-I/AGS-SI-I (3).jpg]

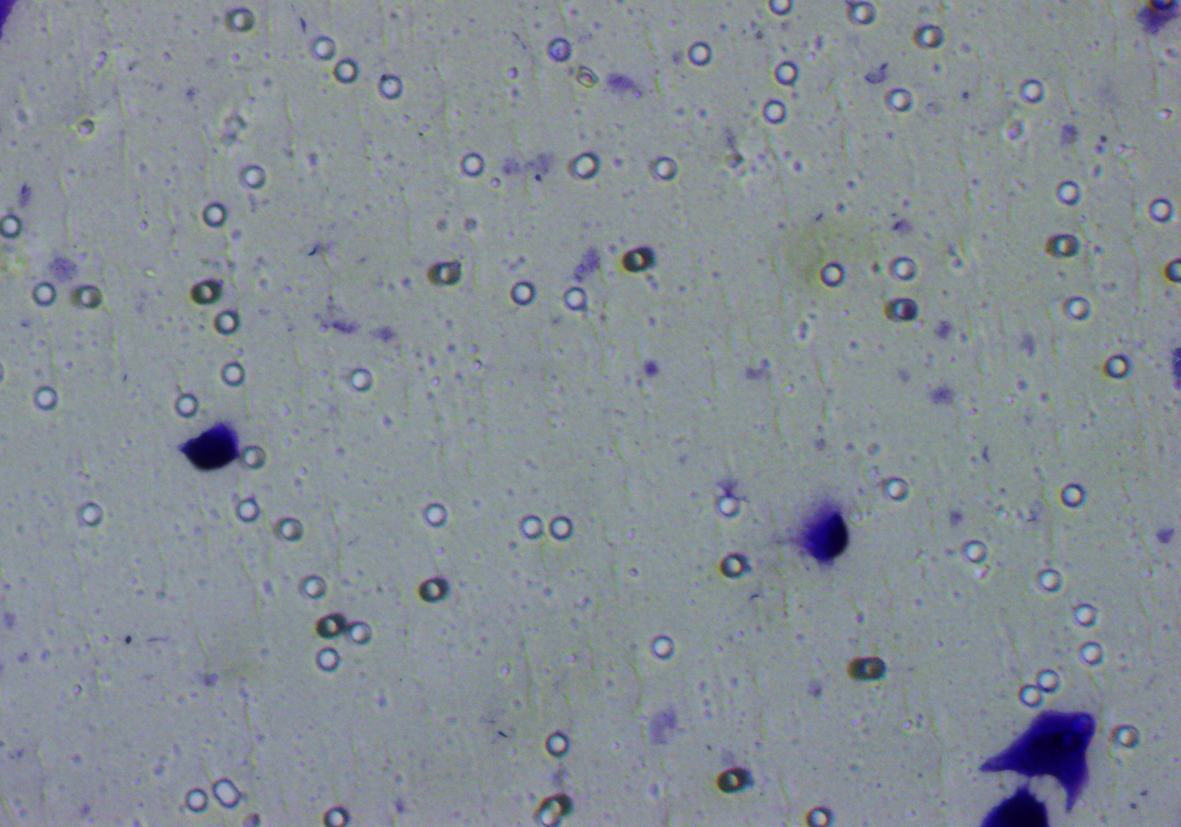

Supplement: Supplemental Information 1 [file peerj-12-17082-s001.zip › AGS-SI-I/AGS-SI-I (4).jpg]

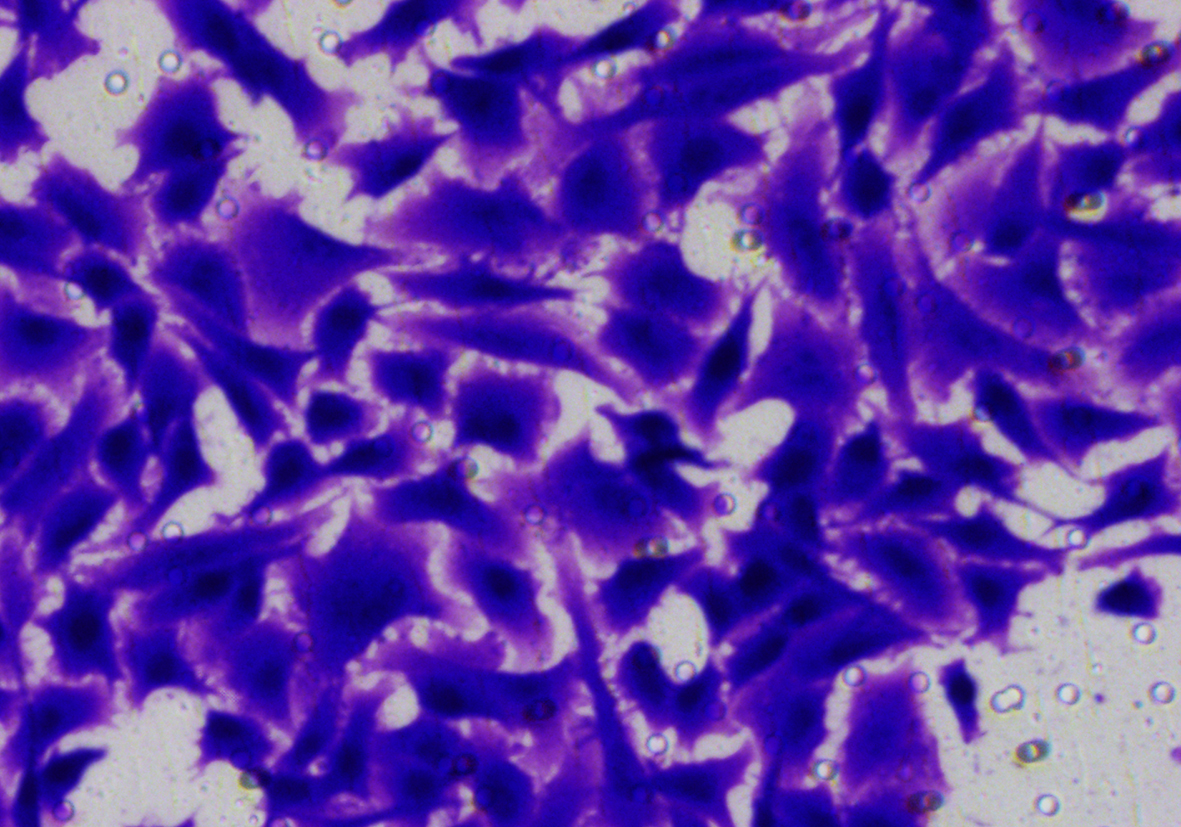

Supplement: Supplemental Information 2 [file peerj-12-17082-s002.zip › AGS-NC-M/AGS-NC-M (1).jpg]

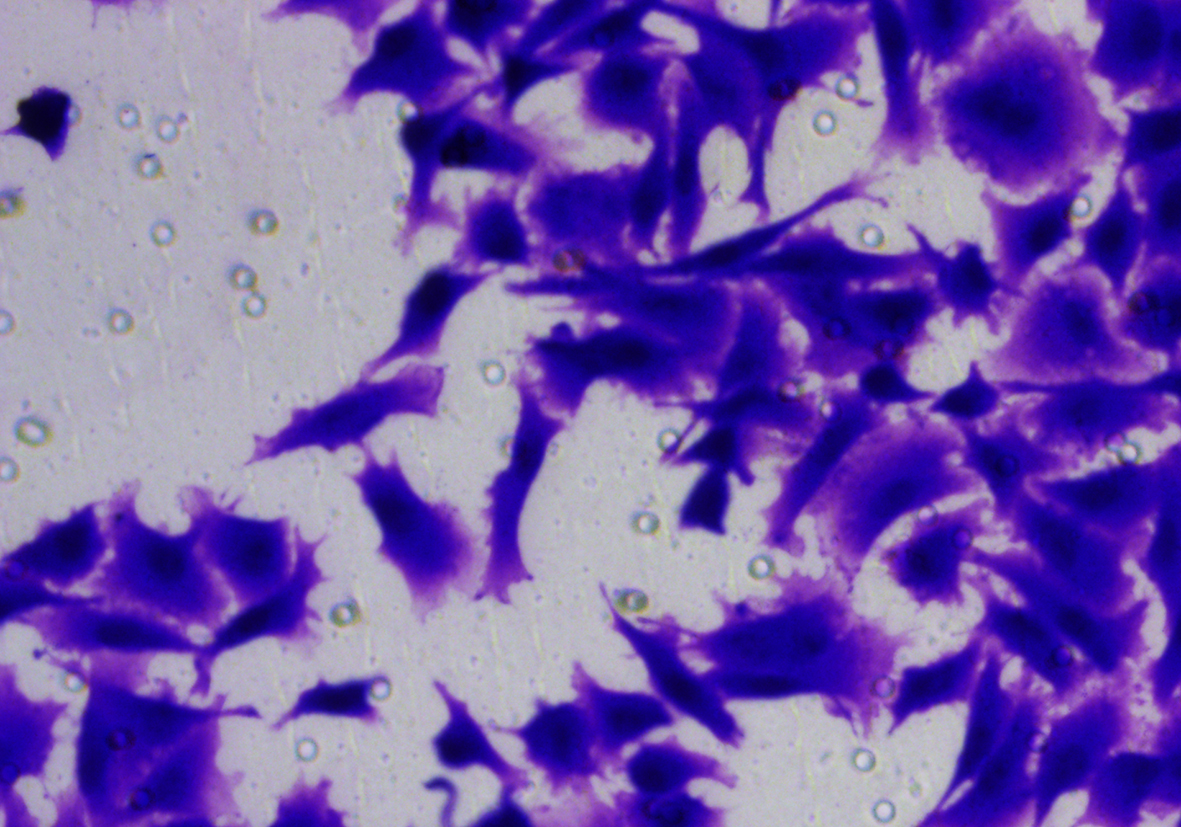

Supplement: Supplemental Information 2 [file peerj-12-17082-s002.zip › AGS-NC-M/AGS-NC-M (2).jpg]

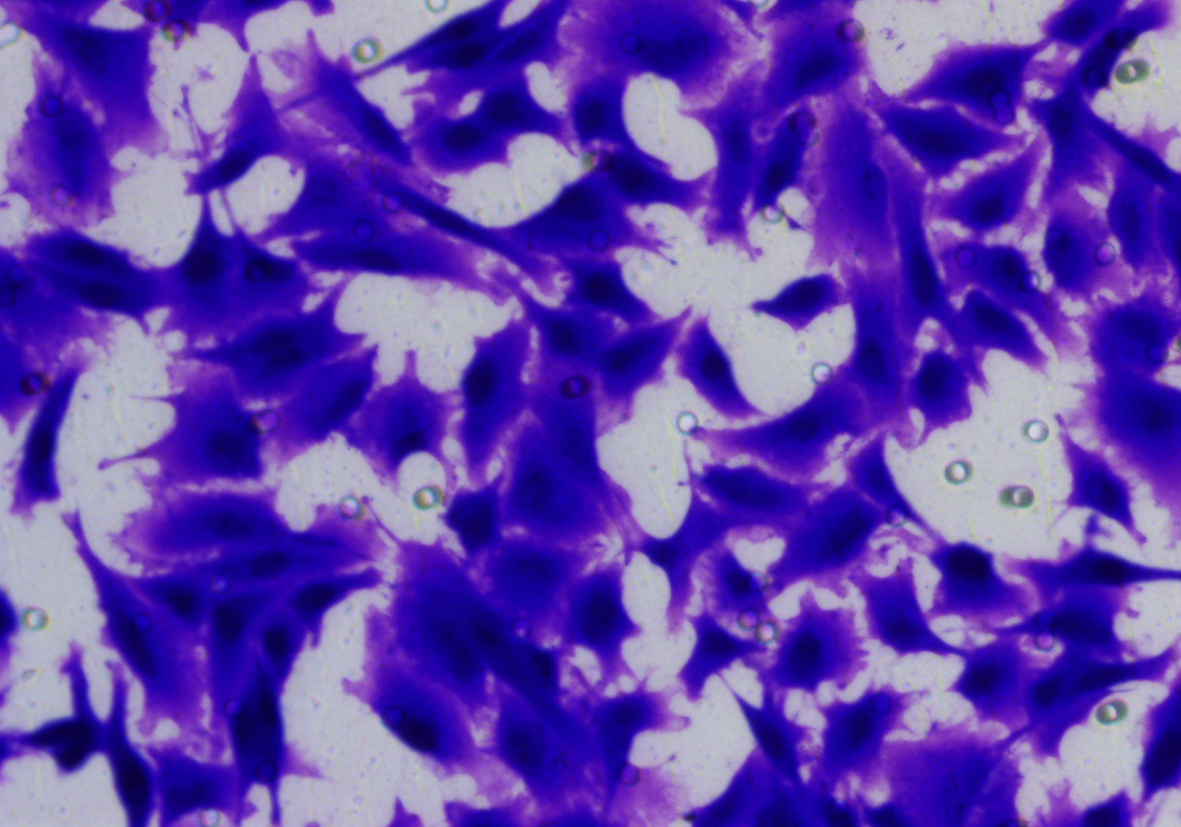

Supplement: Supplemental Information 2 [file peerj-12-17082-s002.zip › AGS-NC-M/AGS-NC-M (3).jpg]

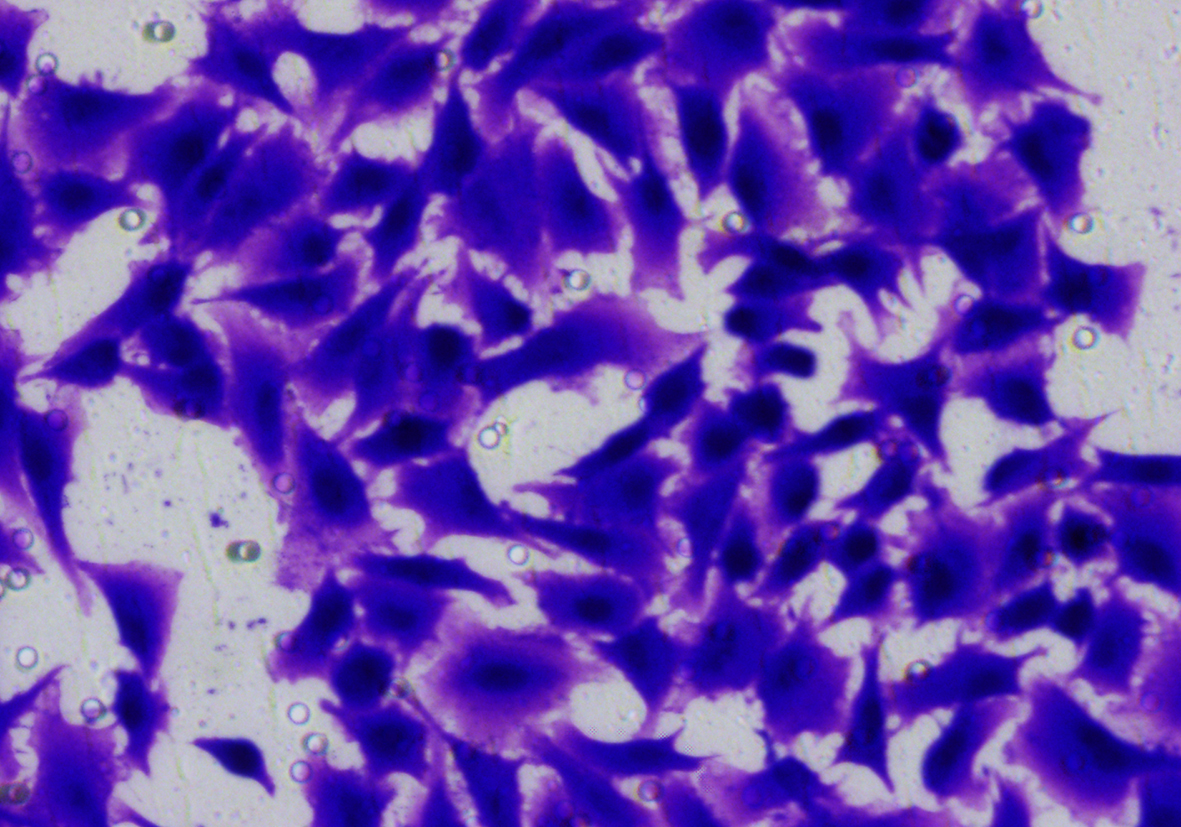

Supplement: Supplemental Information 2 [file peerj-12-17082-s002.zip › AGS-NC-M/AGS-NC-M (4).jpg]

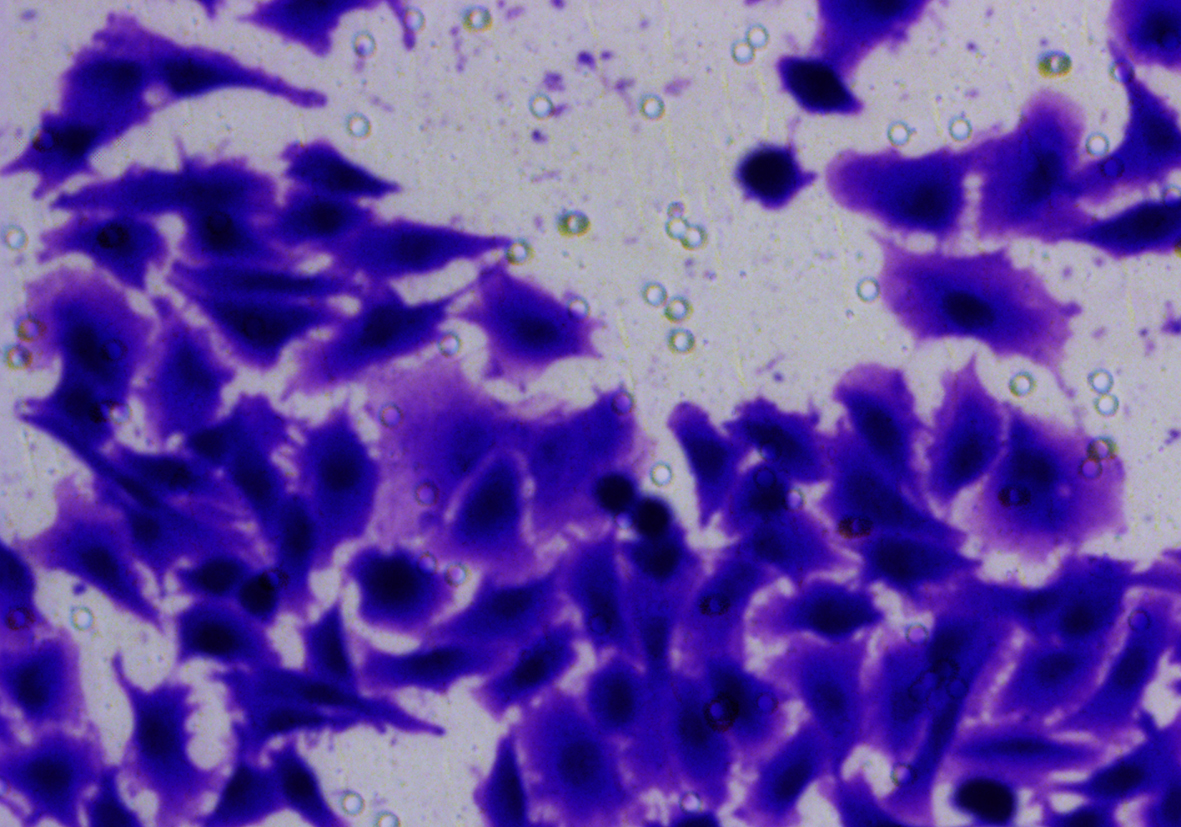

Supplement: Supplemental Information 3 [file peerj-12-17082-s003.zip › AGS-NC-I/AGS-NC-I (1).jpg]

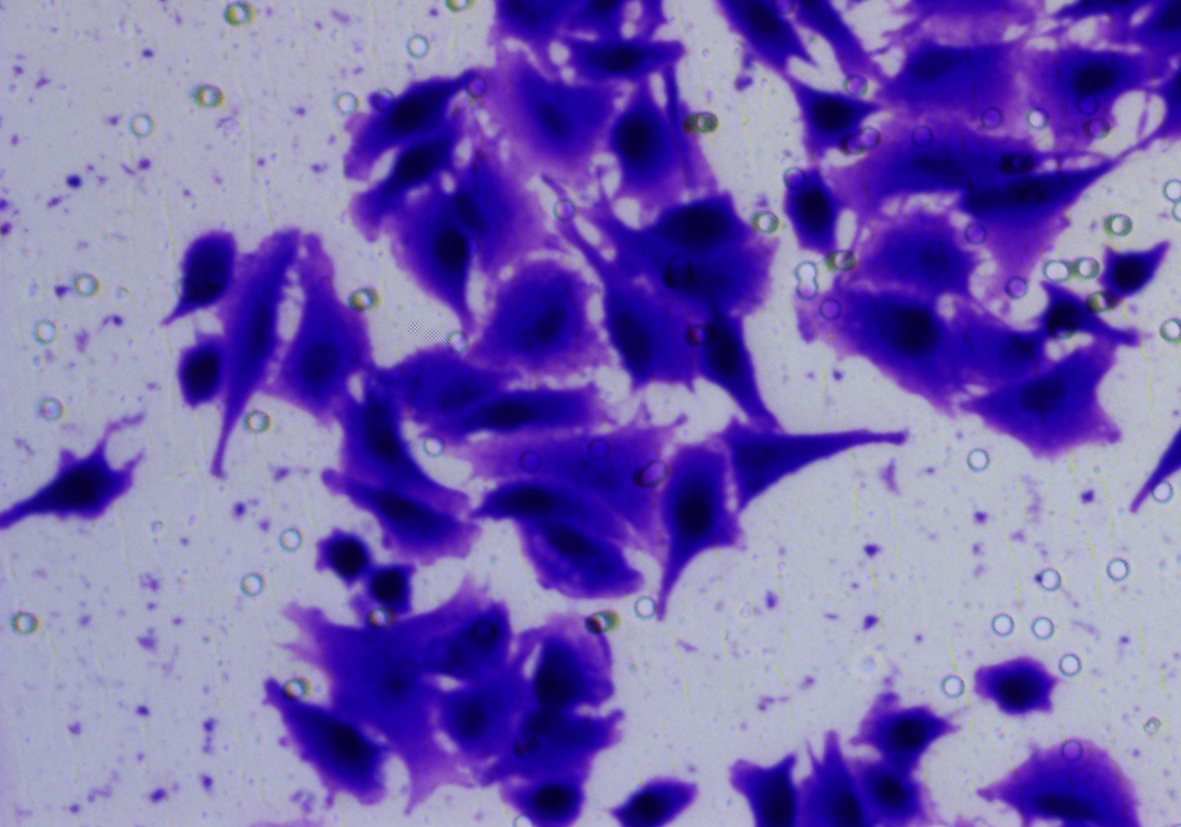

Supplement: Supplemental Information 3 [file peerj-12-17082-s003.zip › AGS-NC-I/AGS-NC-I (2).jpg]

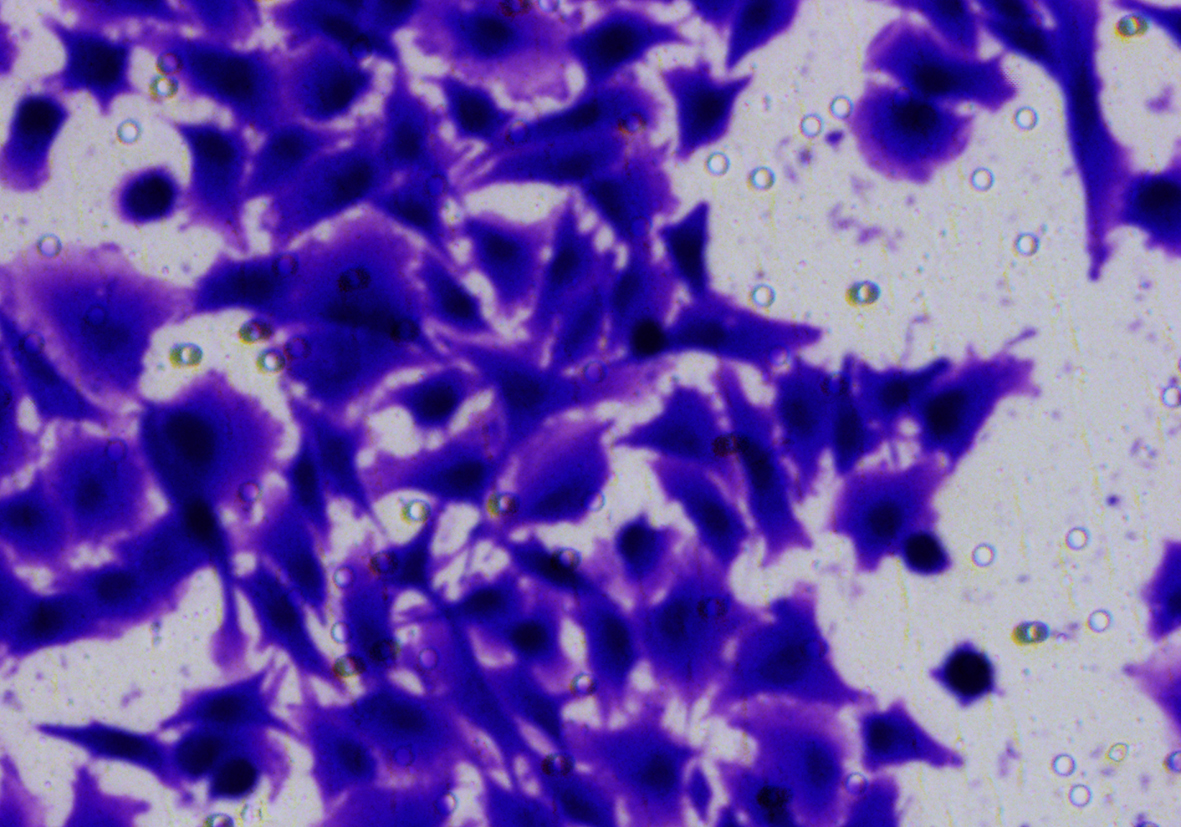

Supplement: Supplemental Information 3 [file peerj-12-17082-s003.zip › AGS-NC-I/AGS-NC-I (3).jpg]

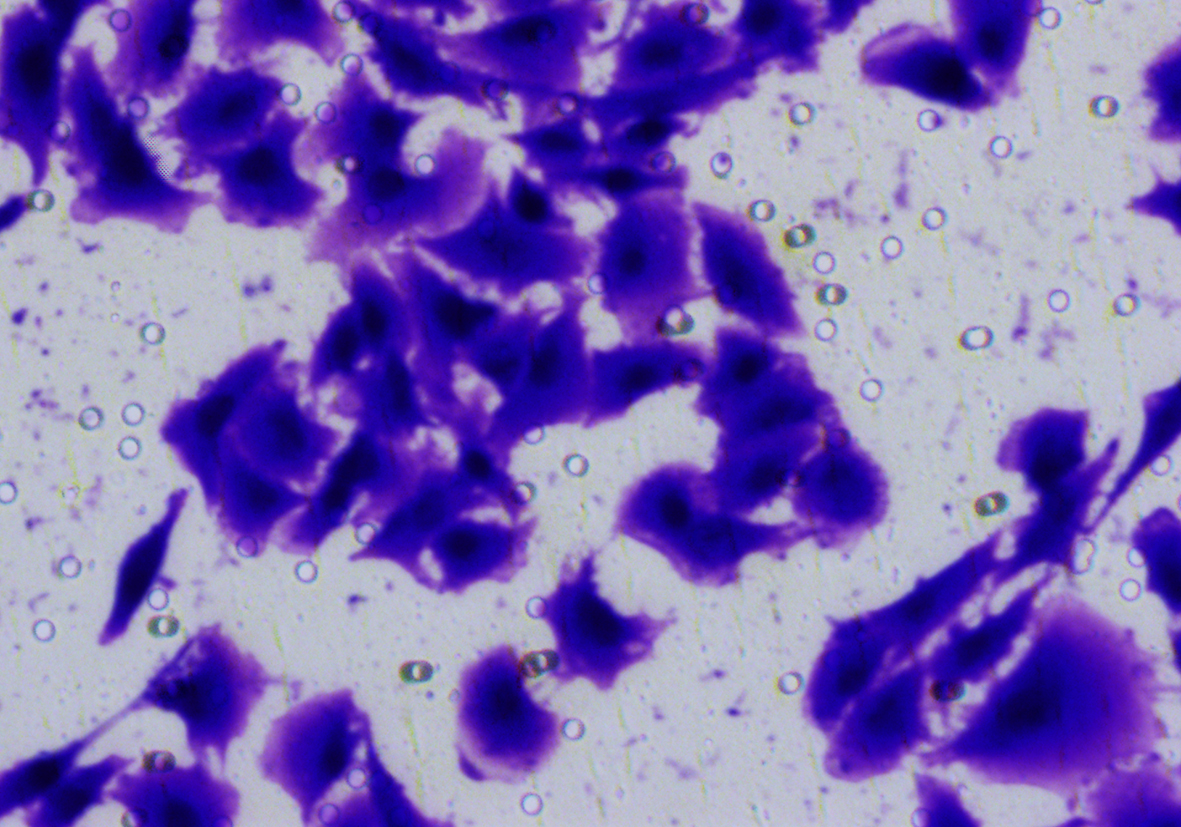

Supplement: Supplemental Information 3 [file peerj-12-17082-s003.zip › AGS-NC-I/AGS-NC-I (4).jpg]

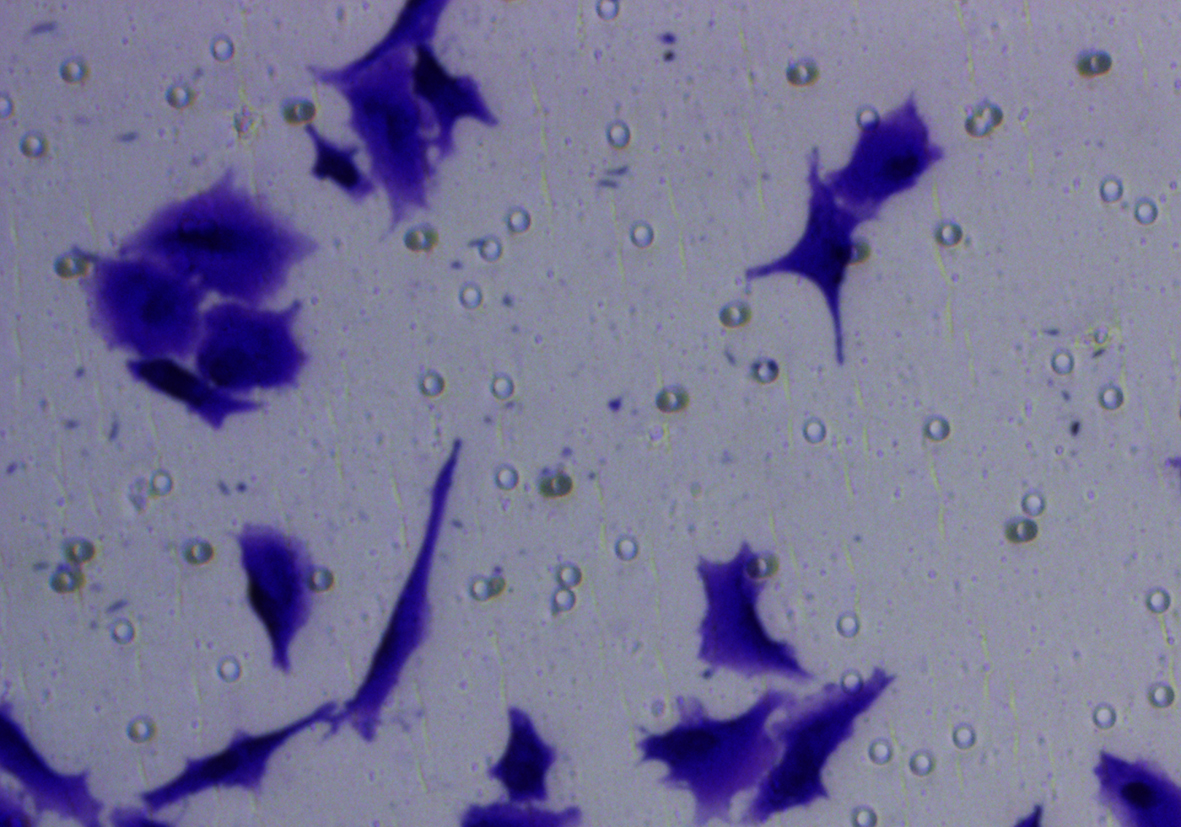

Supplement: Supplemental Information 4 [file peerj-12-17082-s004.zip › AGS-SI-M/AGS-SI-M (1).jpg]

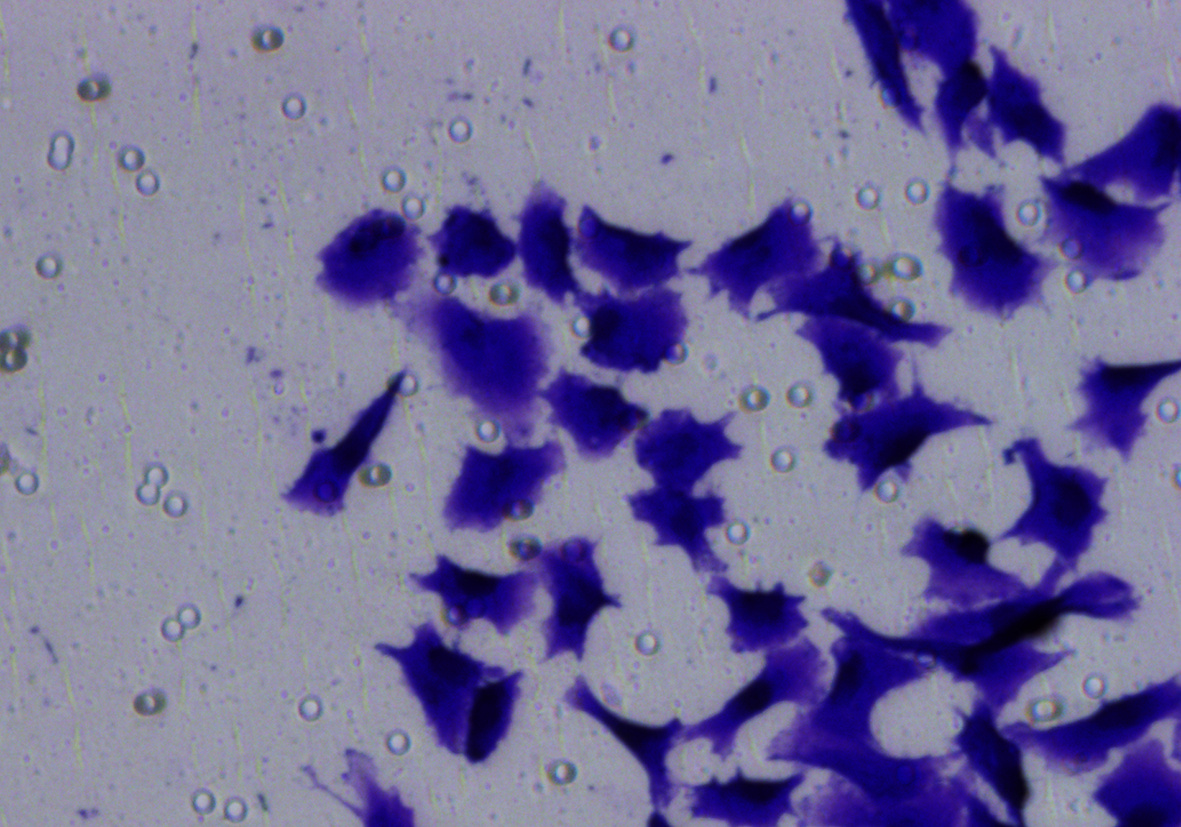

Supplement: Supplemental Information 4 [file peerj-12-17082-s004.zip › AGS-SI-M/AGS-SI-M (2).jpg]

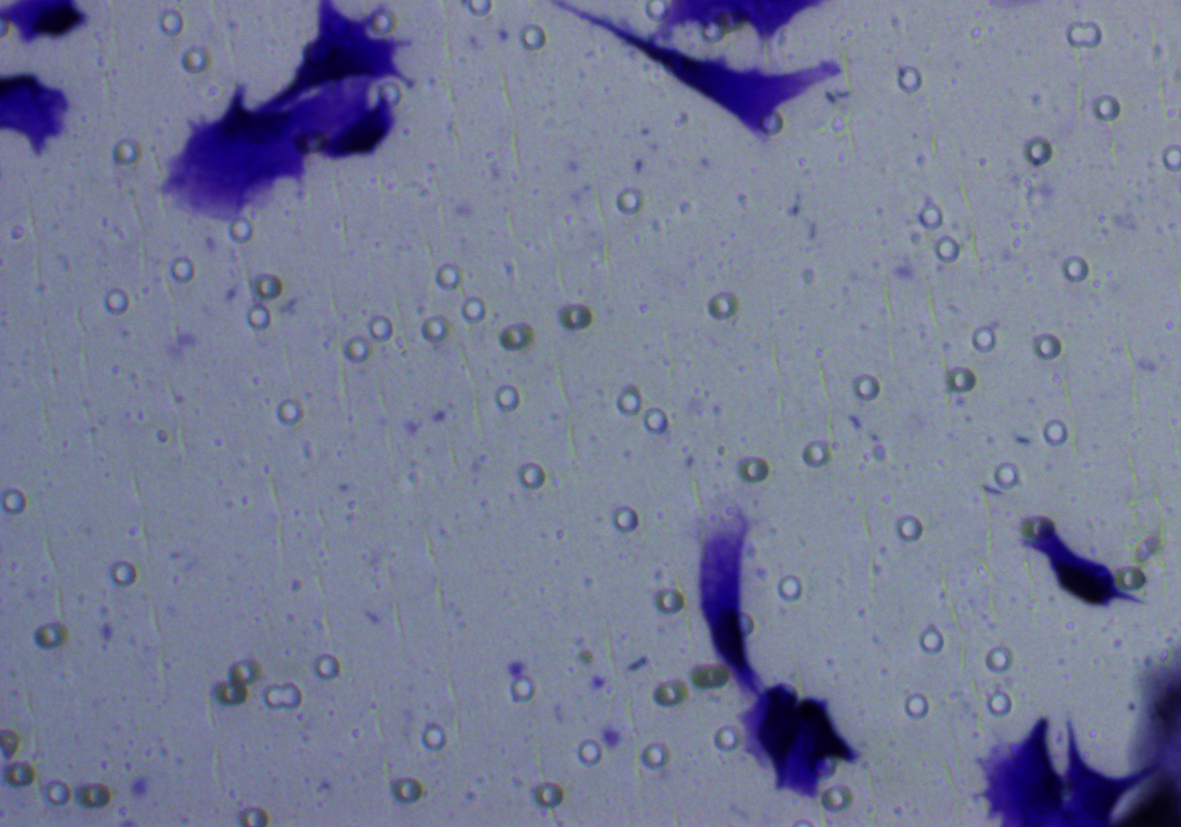

Supplement: Supplemental Information 4 [file peerj-12-17082-s004.zip › AGS-SI-M/AGS-SI-M (3).jpg]

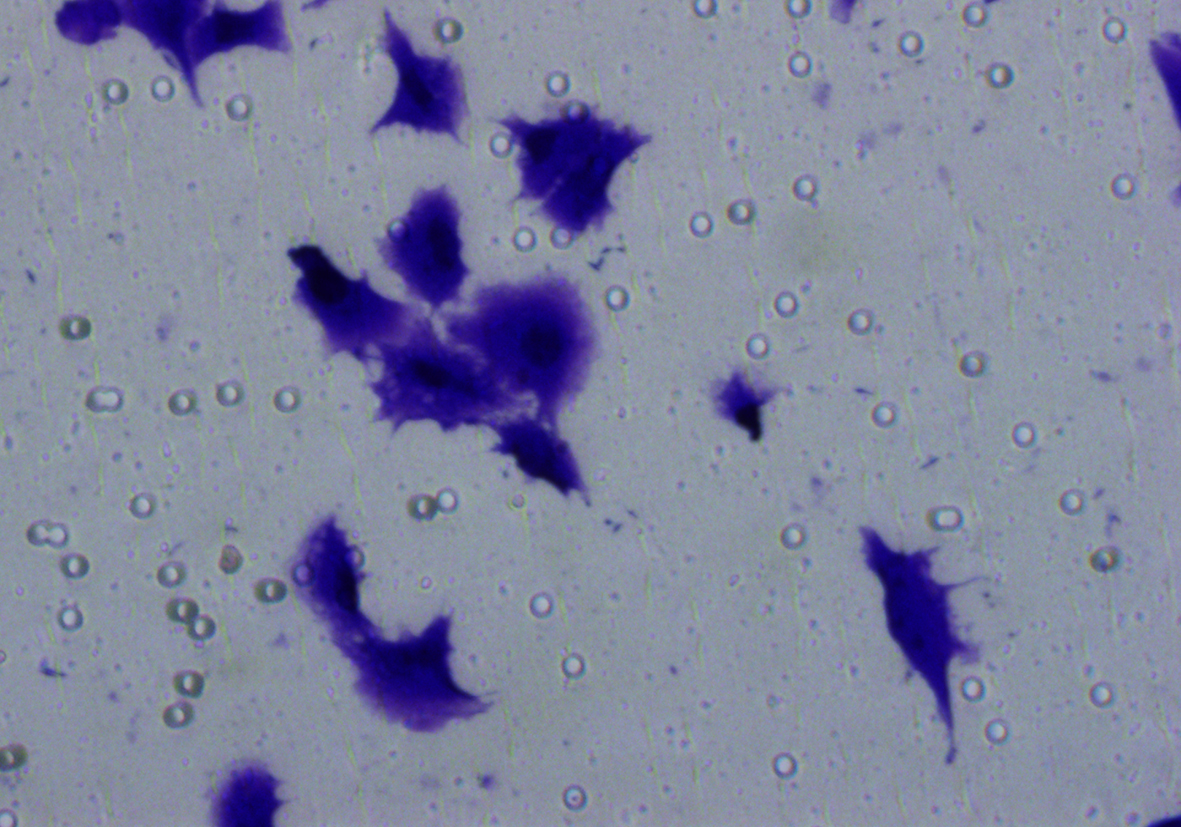

Supplement: Supplemental Information 4 [file peerj-12-17082-s004.zip › AGS-SI-M/AGS-SI-M (4).jpg]

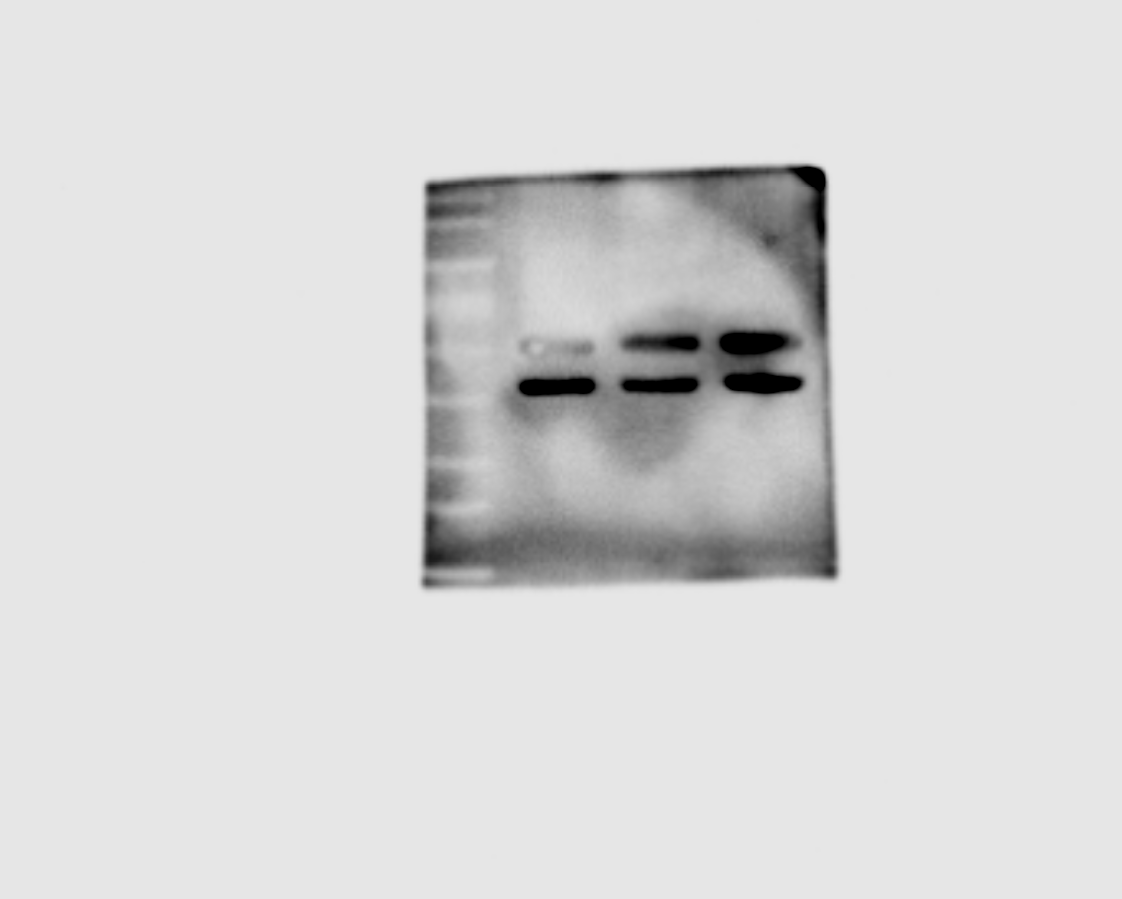

Supplement: Supplemental Information 5 [file peerj-12-17082-s005.zip › raw data/WB/pparg 1_1(Chemiluminescence).tif]

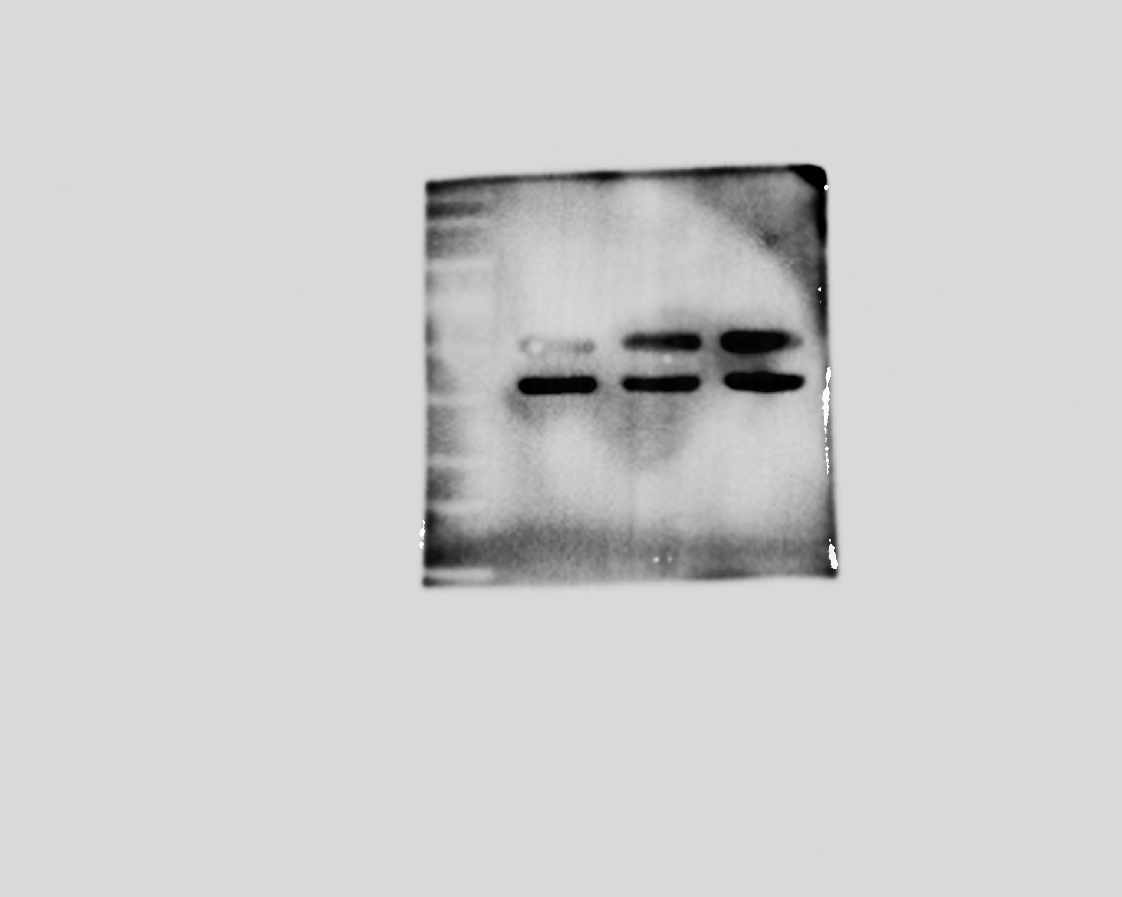

Supplement: Supplemental Information 5 [file peerj-12-17082-s005.zip › raw data/WB/pparg 1_3(Composite).tif]

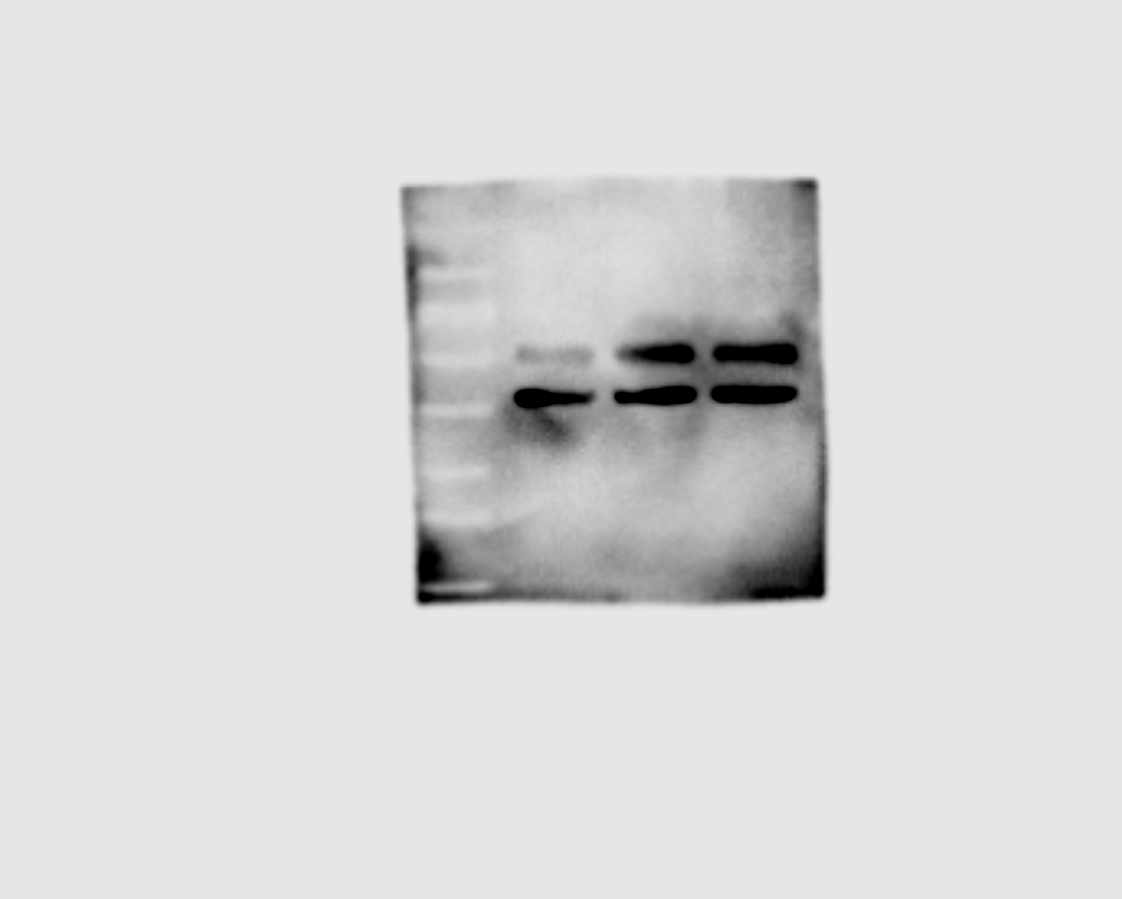

Supplement: Supplemental Information 5 [file peerj-12-17082-s005.zip › raw data/WB/pparg 2_1(Chemiluminescence).tif]

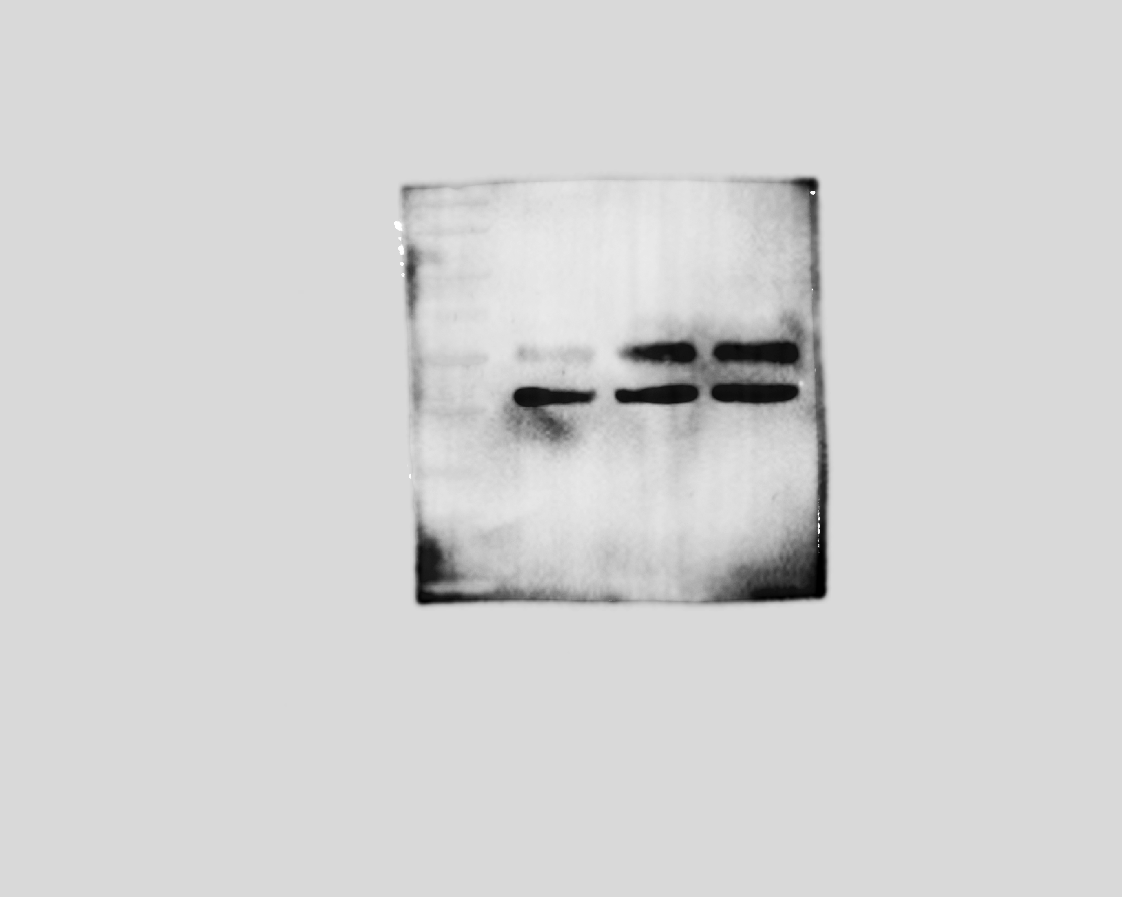

Supplement: Supplemental Information 5 [file peerj-12-17082-s005.zip › raw data/WB/pparg 2_3(Composite).tif]

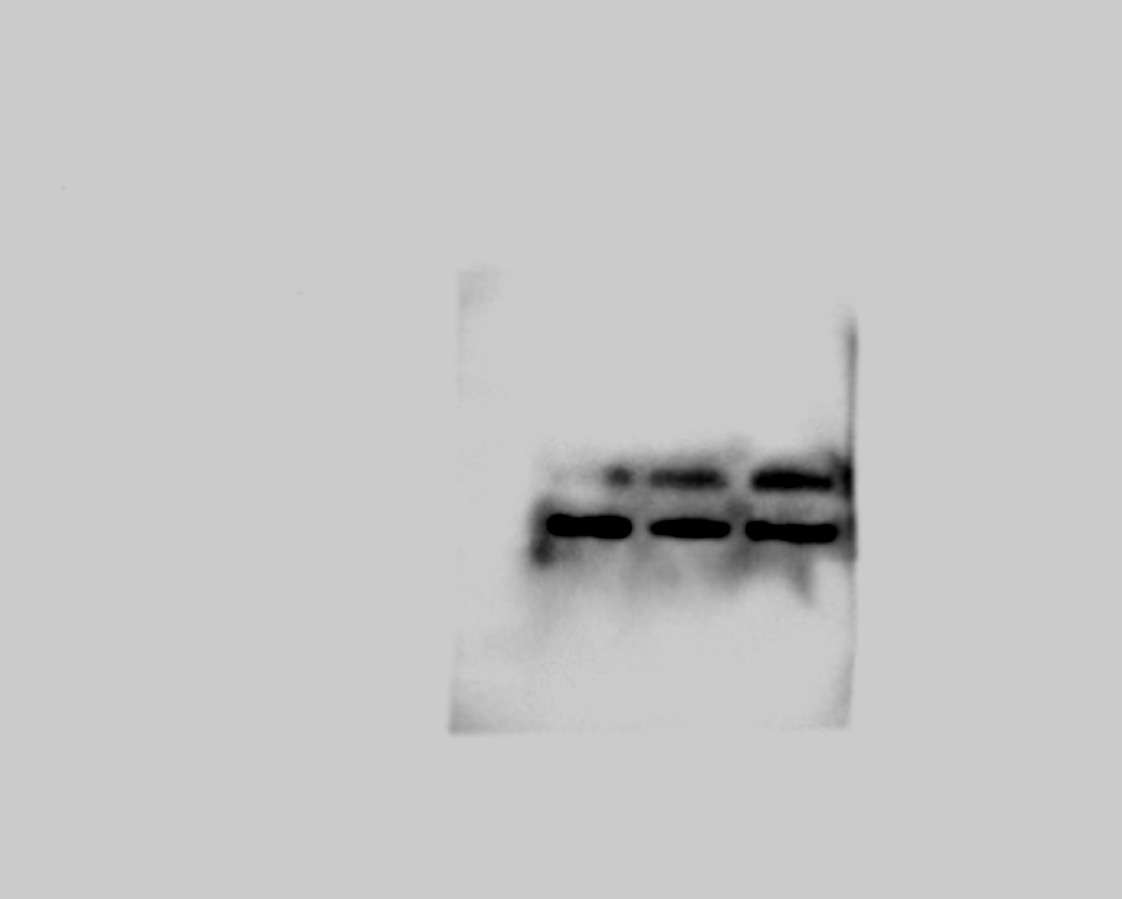

Supplement: Supplemental Information 5 [file peerj-12-17082-s005.zip › raw data/WB/pparg 3_1(Chemiluminescence).tif]

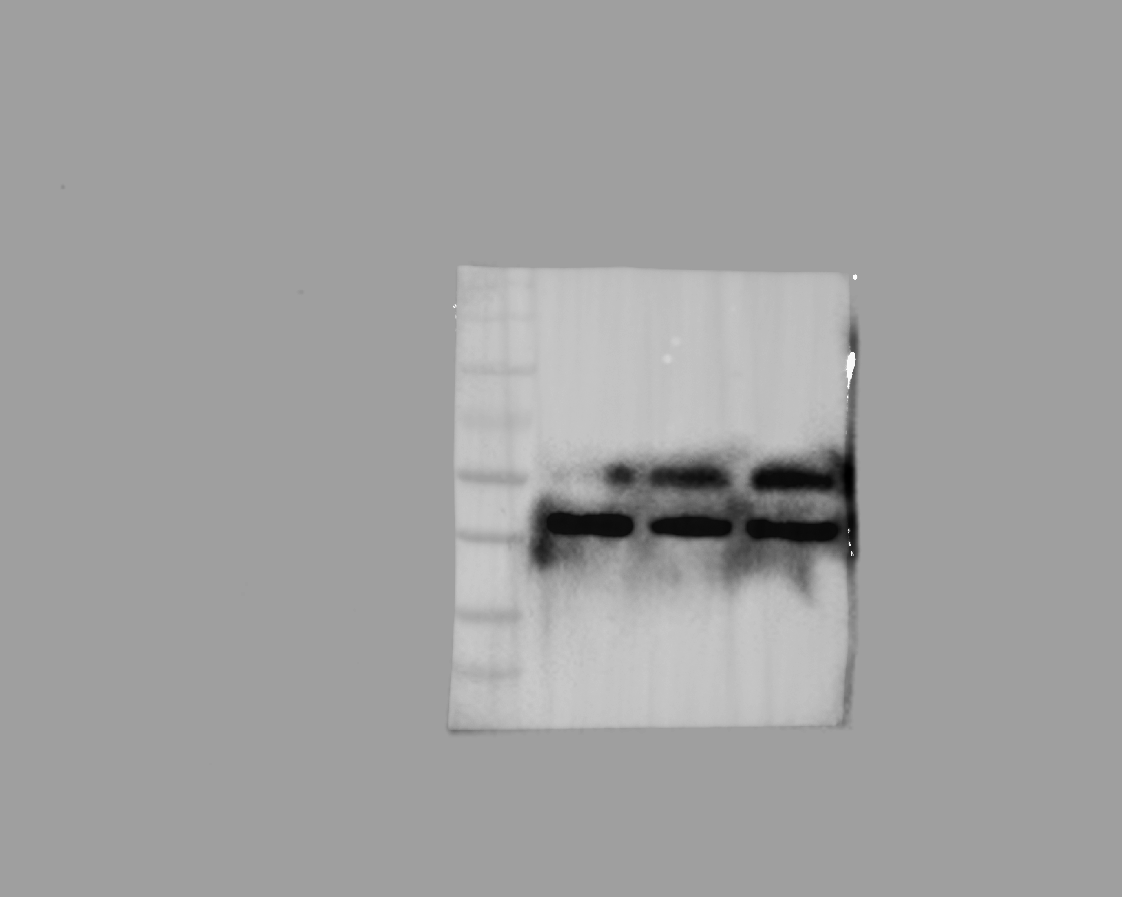

Supplement: Supplemental Information 5 [file peerj-12-17082-s005.zip › raw data/WB/pparg 3_3(Composite).tif]

# Repeat 1

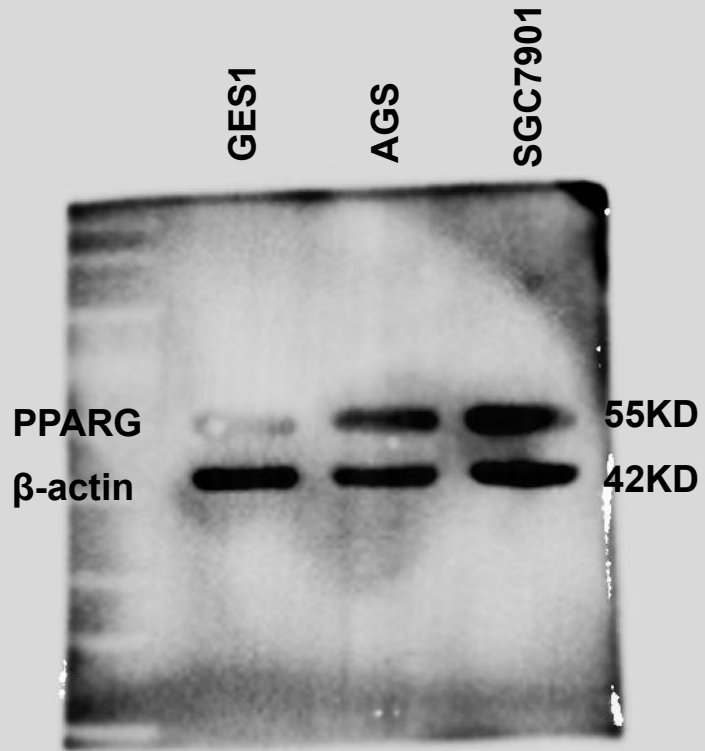

## Repeat 2

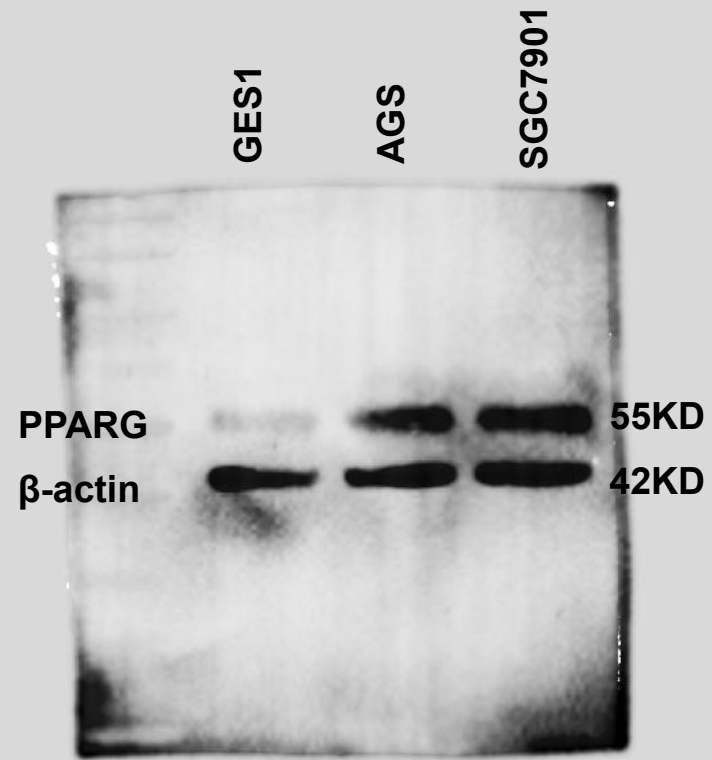

# Repeat 3

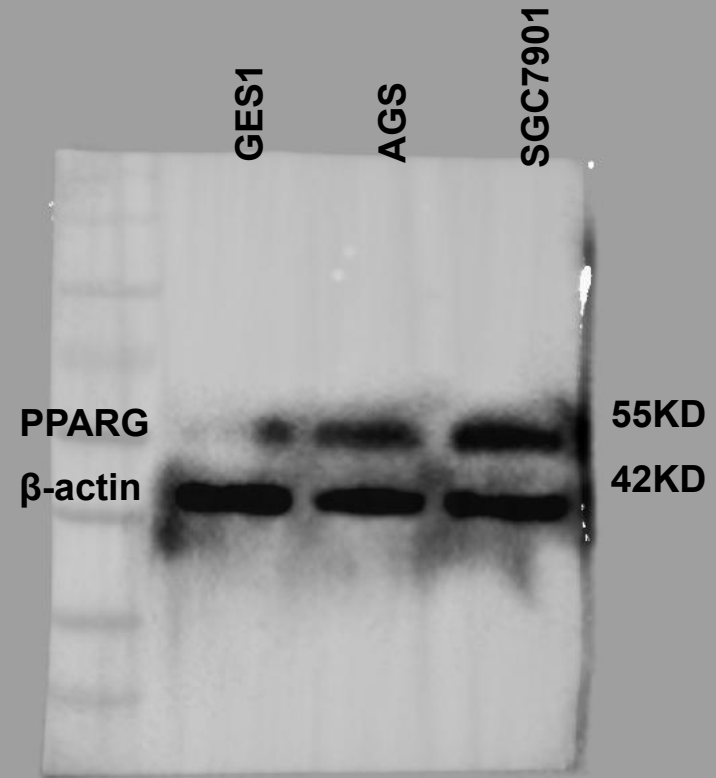

Supplement: Supplemental Information 5 [file peerj-12-17082-s005.zip › raw data/WB raw data.pdf]

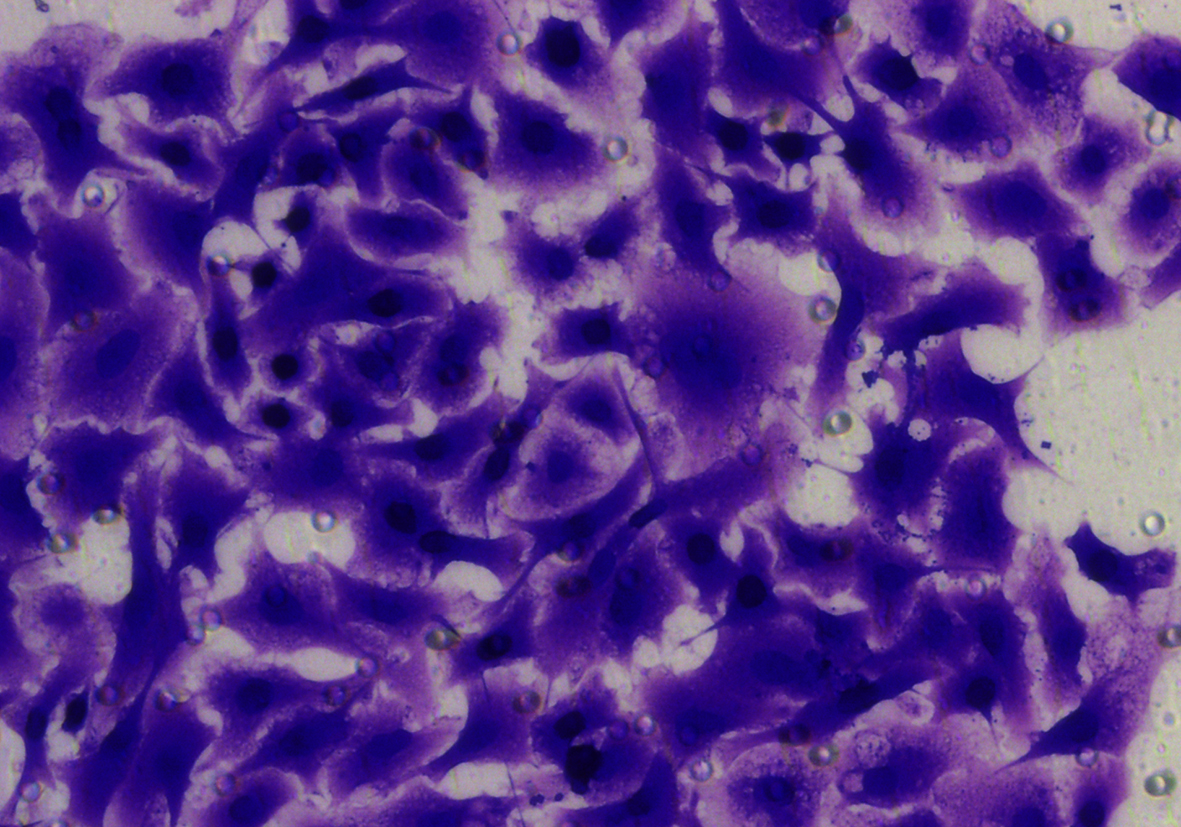

Supplement: Supplemental Information 5 [file peerj-12-17082-s005.zip › raw data/cell numbers/SGC-7901-NC-I/SGC7901-NC-I (1).jpg]

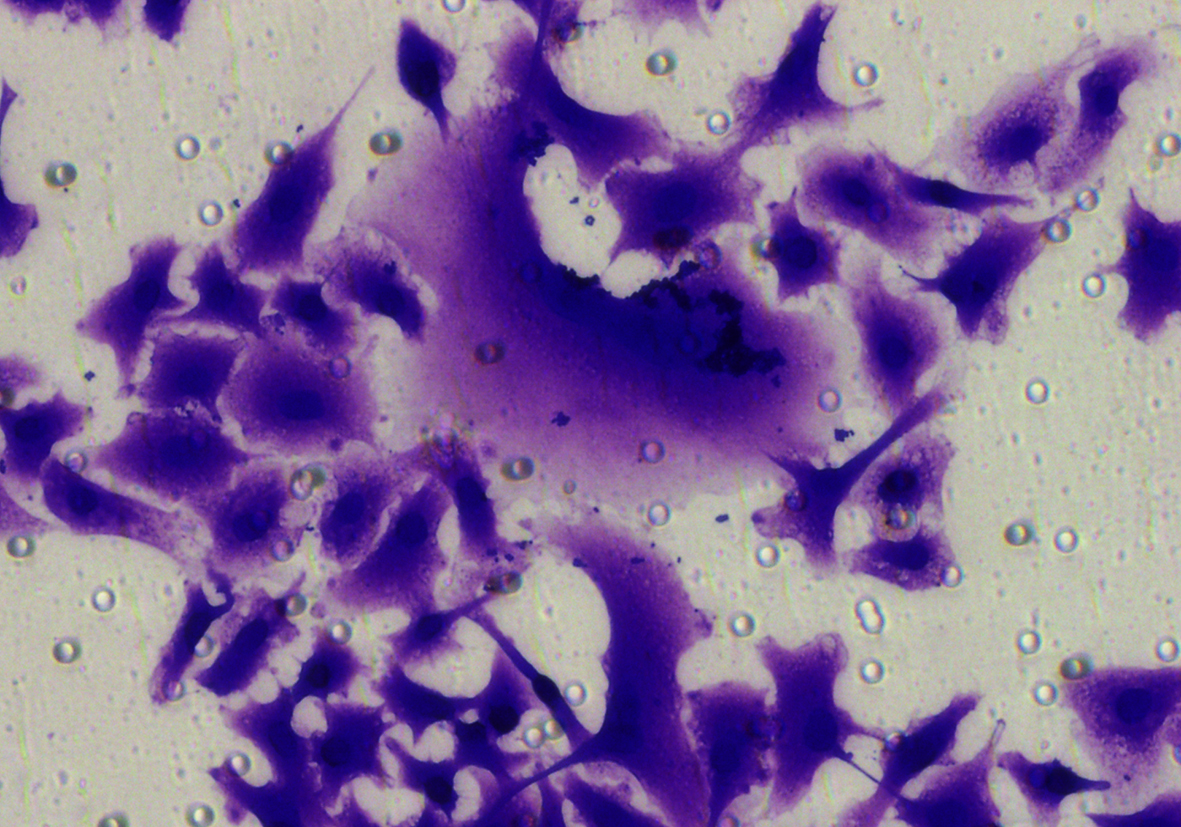

Supplement: Supplemental Information 5 [file peerj-12-17082-s005.zip › raw data/cell numbers/SGC-7901-NC-I/SGC7901-NC-I (2).jpg]

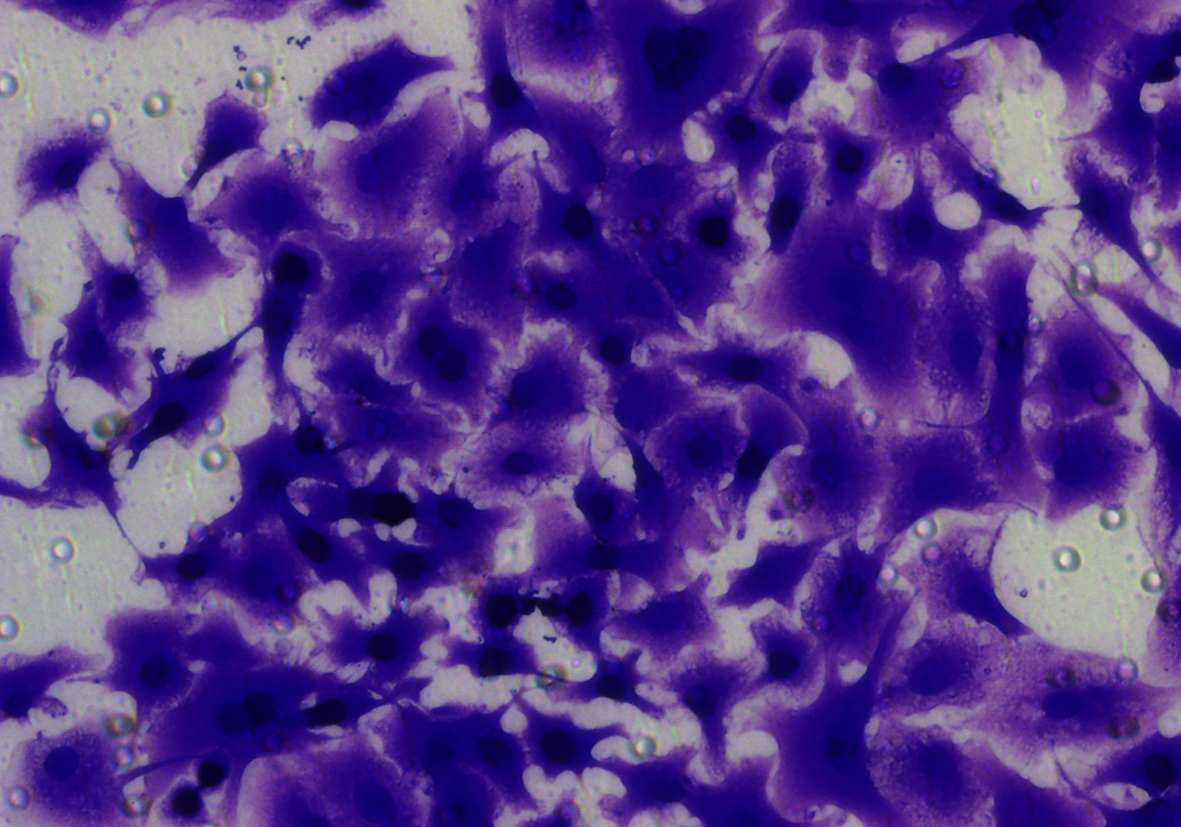

Supplement: Supplemental Information 5 [file peerj-12-17082-s005.zip › raw data/cell numbers/SGC-7901-NC-I/SGC7901-NC-I (3).jpg]

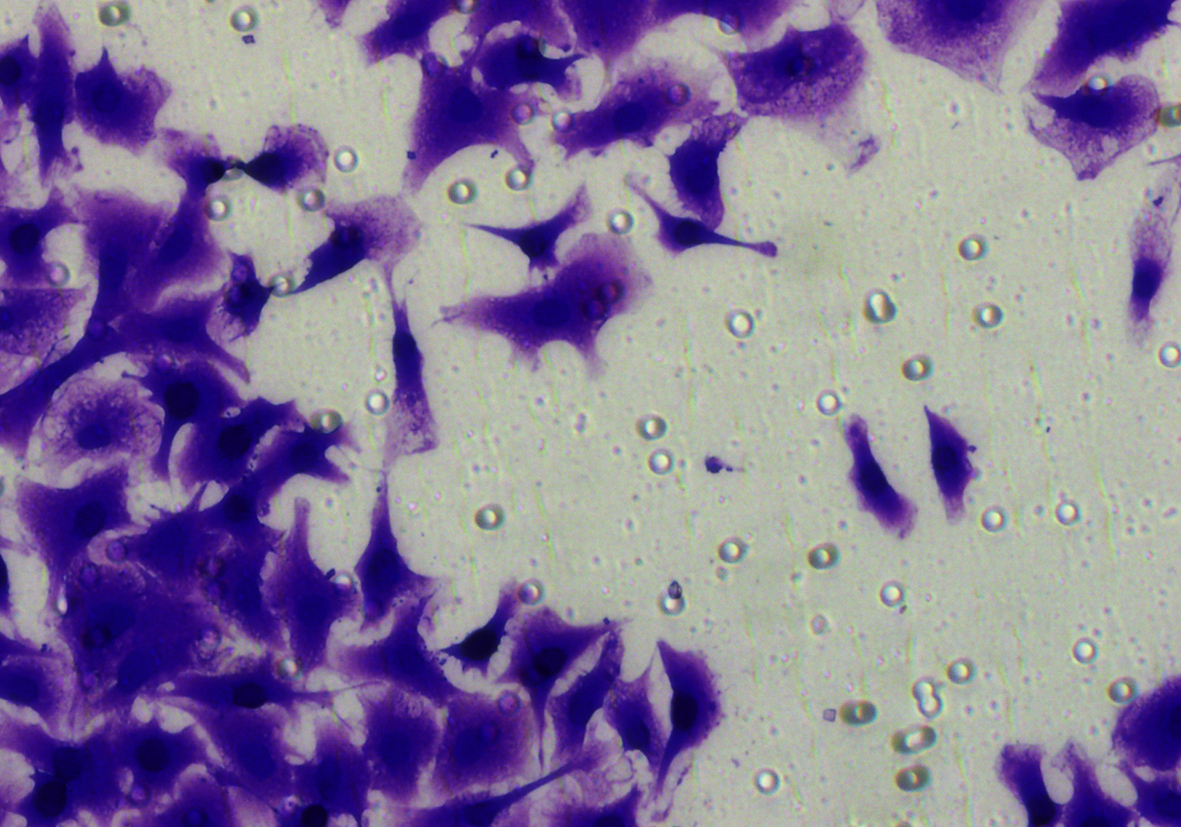

Supplement: Supplemental Information 5 [file peerj-12-17082-s005.zip › raw data/cell numbers/SGC-7901-NC-I/SGC7901-NC-I (4).jpg]

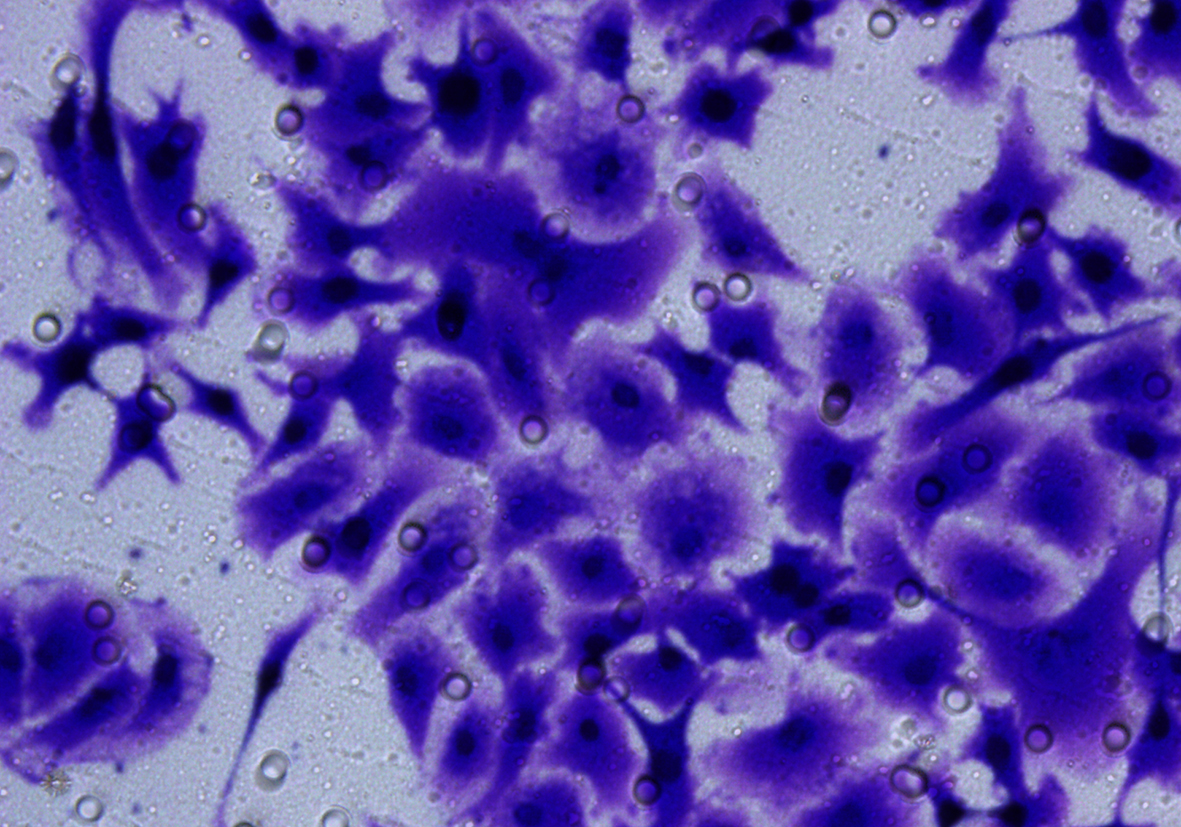

Supplement: Supplemental Information 5 [file peerj-12-17082-s005.zip › raw data/cell numbers/SGC-7901-NC-M/SGC7901-NC-M (1).jpg]

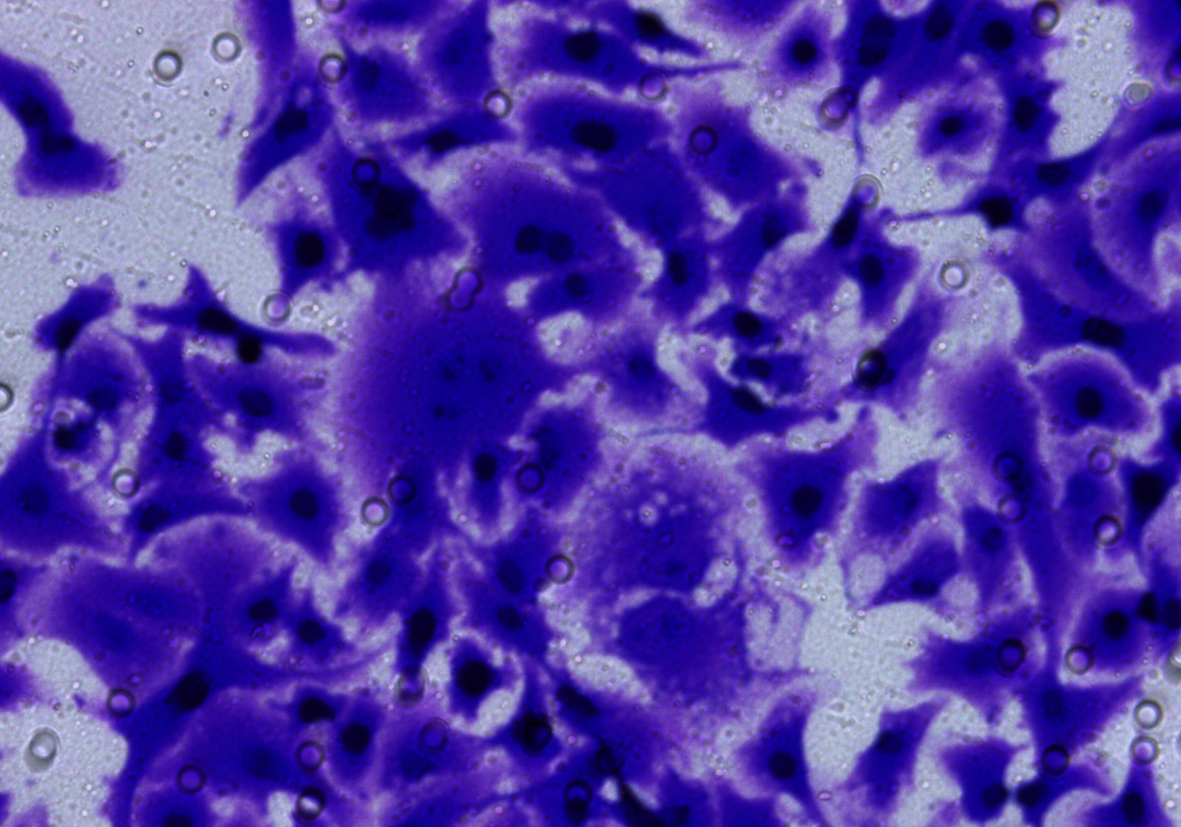

Supplement: Supplemental Information 5 [file peerj-12-17082-s005.zip › raw data/cell numbers/SGC-7901-NC-M/SGC7901-NC-M (2).jpg]

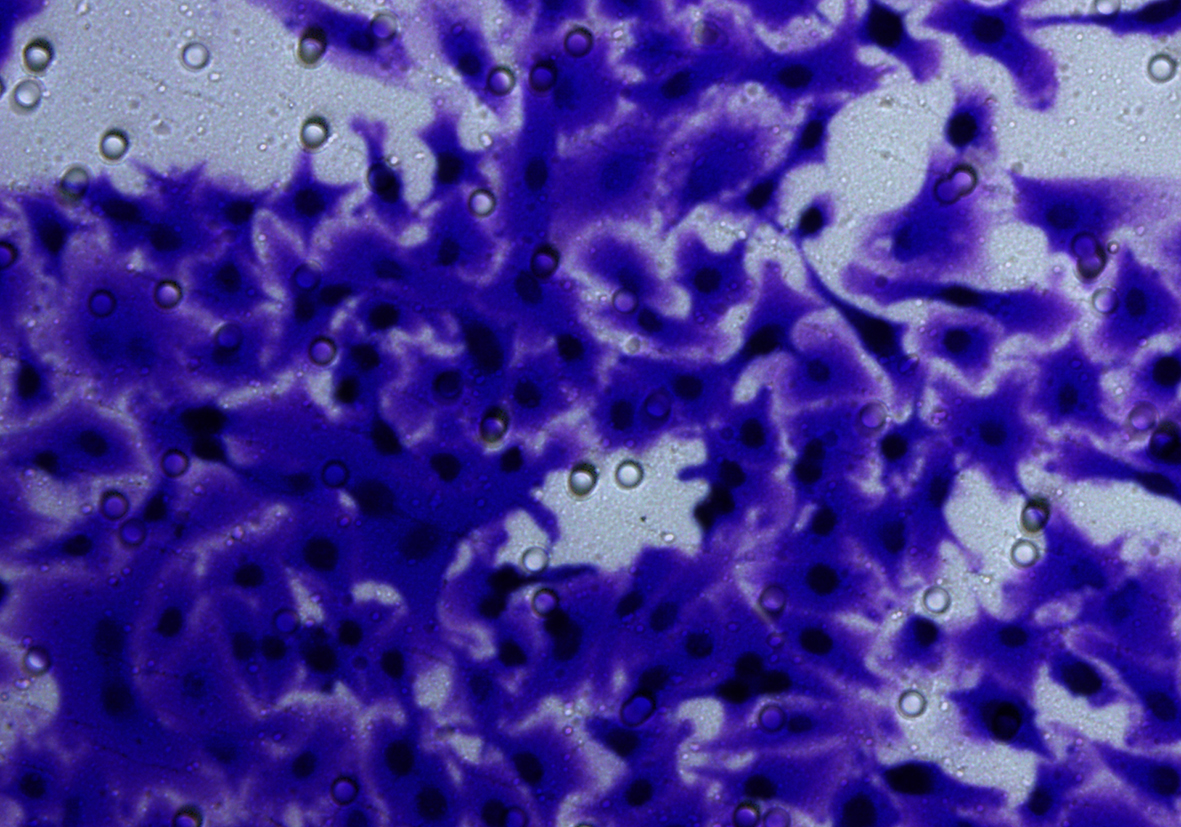

Supplement: Supplemental Information 5 [file peerj-12-17082-s005.zip › raw data/cell numbers/SGC-7901-NC-M/SGC7901-NC-M (3).jpg]

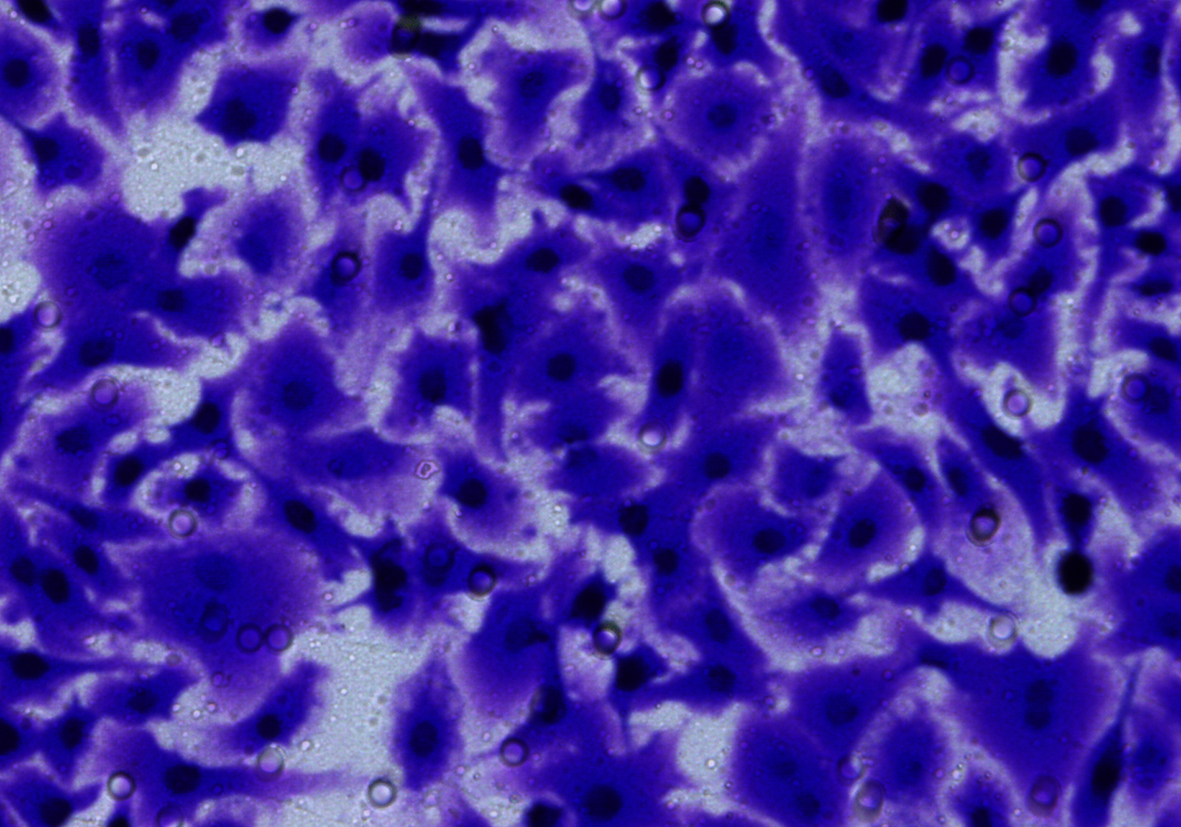

Supplement: Supplemental Information 5 [file peerj-12-17082-s005.zip › raw data/cell numbers/SGC-7901-NC-M/SGC7901-NC-M (4).jpg]

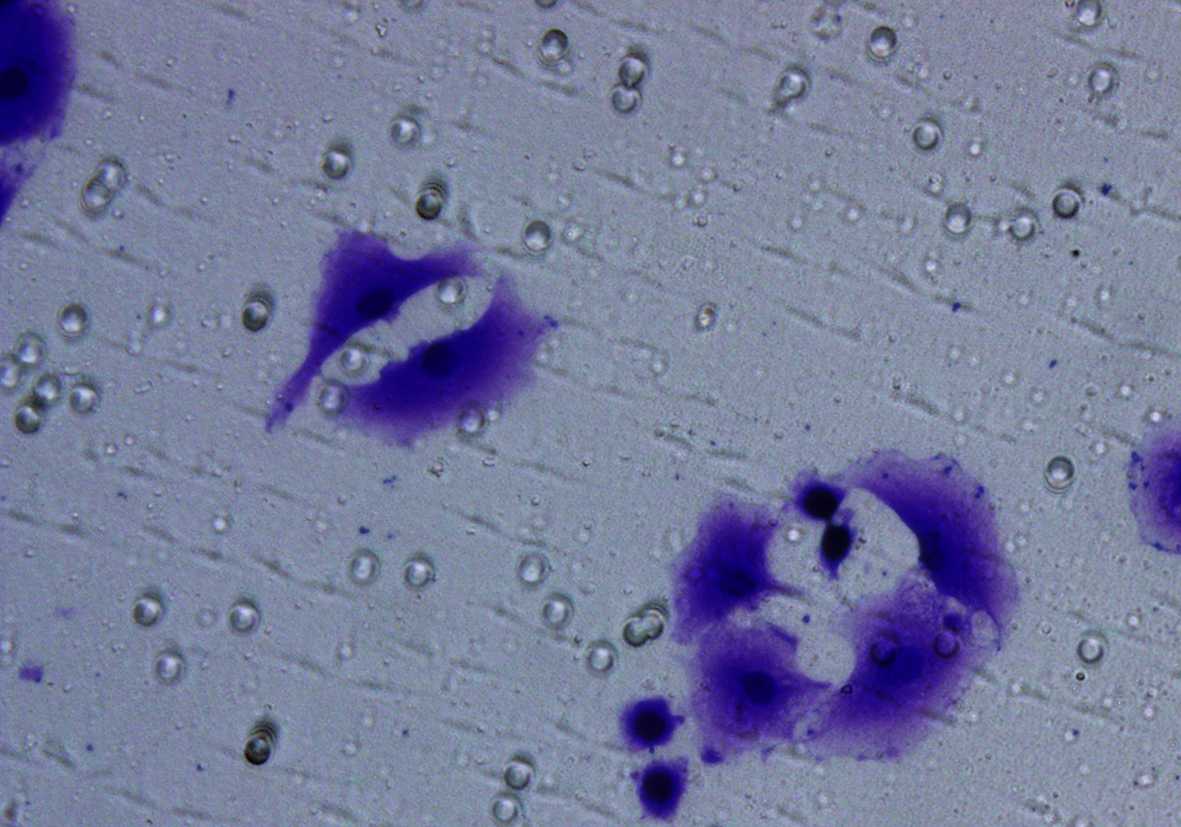

Supplement: Supplemental Information 5 [file peerj-12-17082-s005.zip › raw data/cell numbers/SGC-7901-SI-I/SGC7901-SI-I (1).jpg]

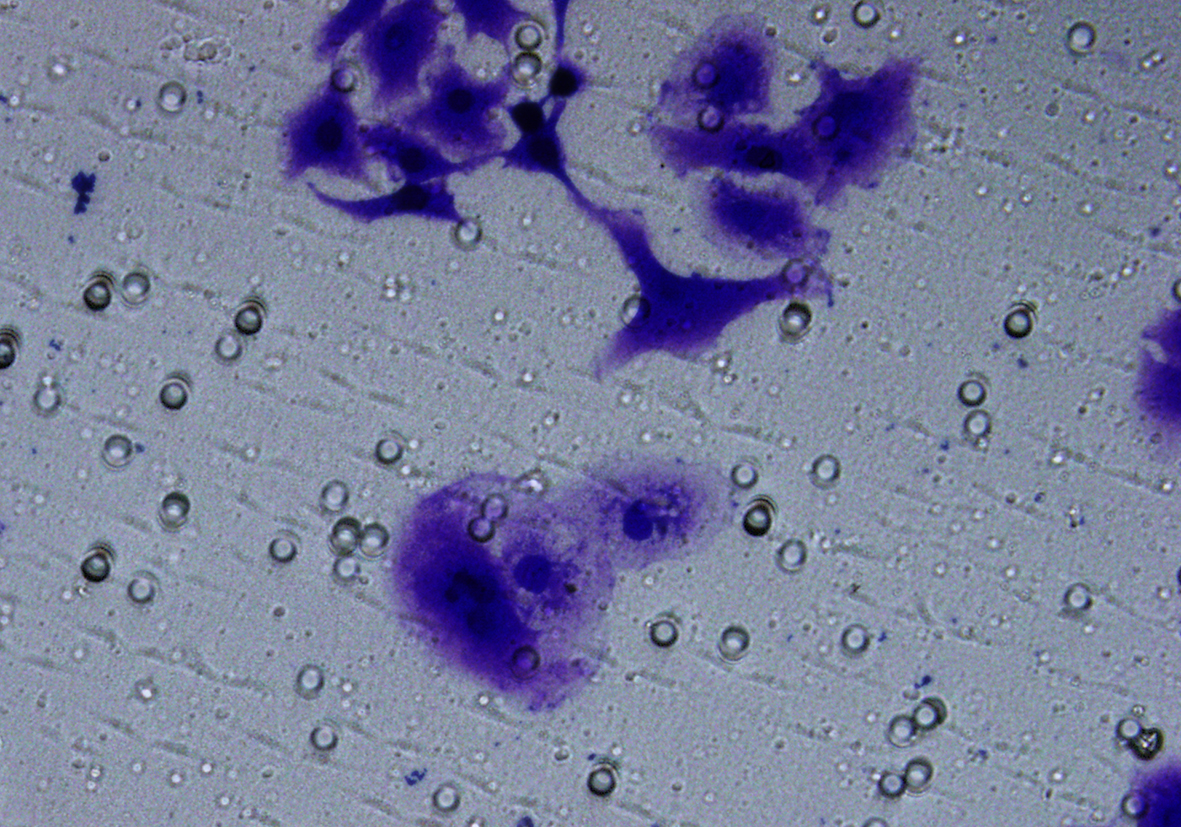

Supplement: Supplemental Information 5 [file peerj-12-17082-s005.zip › raw data/cell numbers/SGC-7901-SI-I/SGC7901-SI-I (2).jpg]

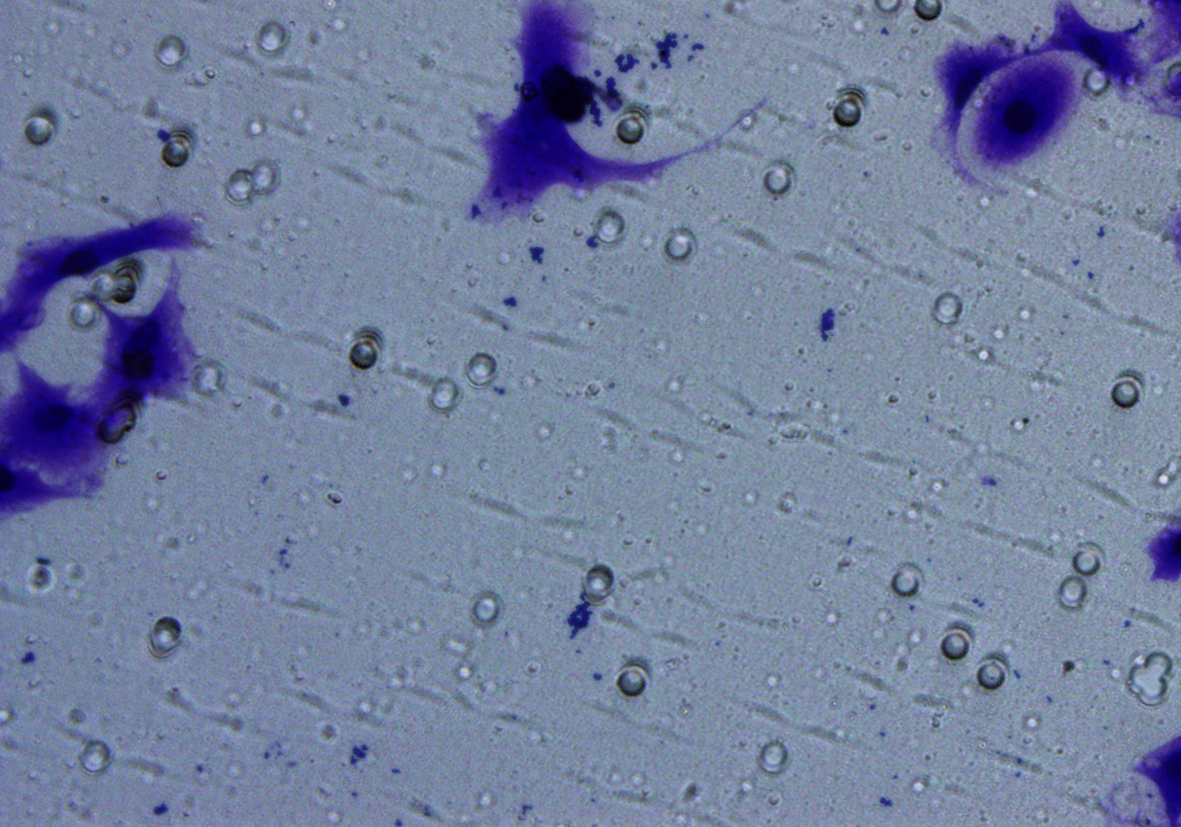

Supplement: Supplemental Information 5 [file peerj-12-17082-s005.zip › raw data/cell numbers/SGC-7901-SI-I/SGC7901-SI-I (3).jpg]

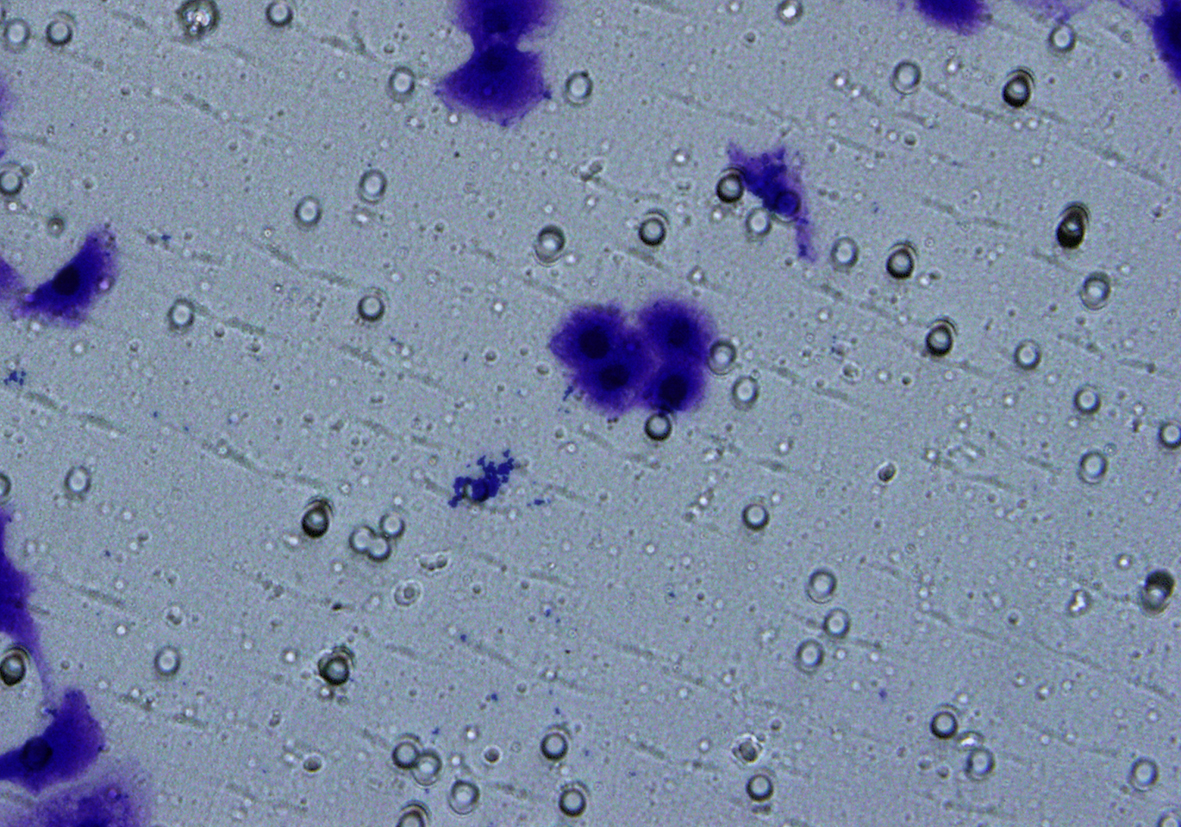

Supplement: Supplemental Information 5 [file peerj-12-17082-s005.zip › raw data/cell numbers/SGC-7901-SI-I/SGC7901-SI-I (4).jpg]

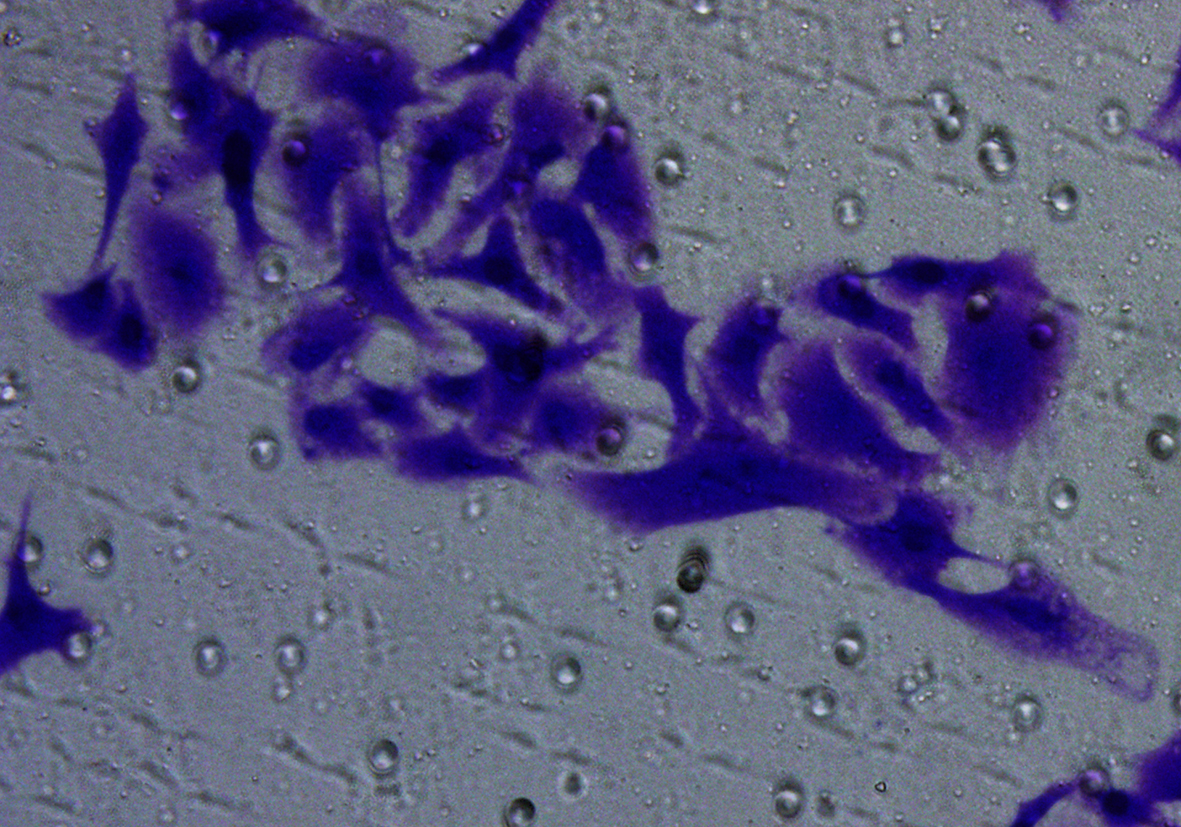

Supplement: Supplemental Information 5 [file peerj-12-17082-s005.zip › raw data/cell numbers/SGC-7901-SI-M/SGC7901-SI-M (1).jpg]

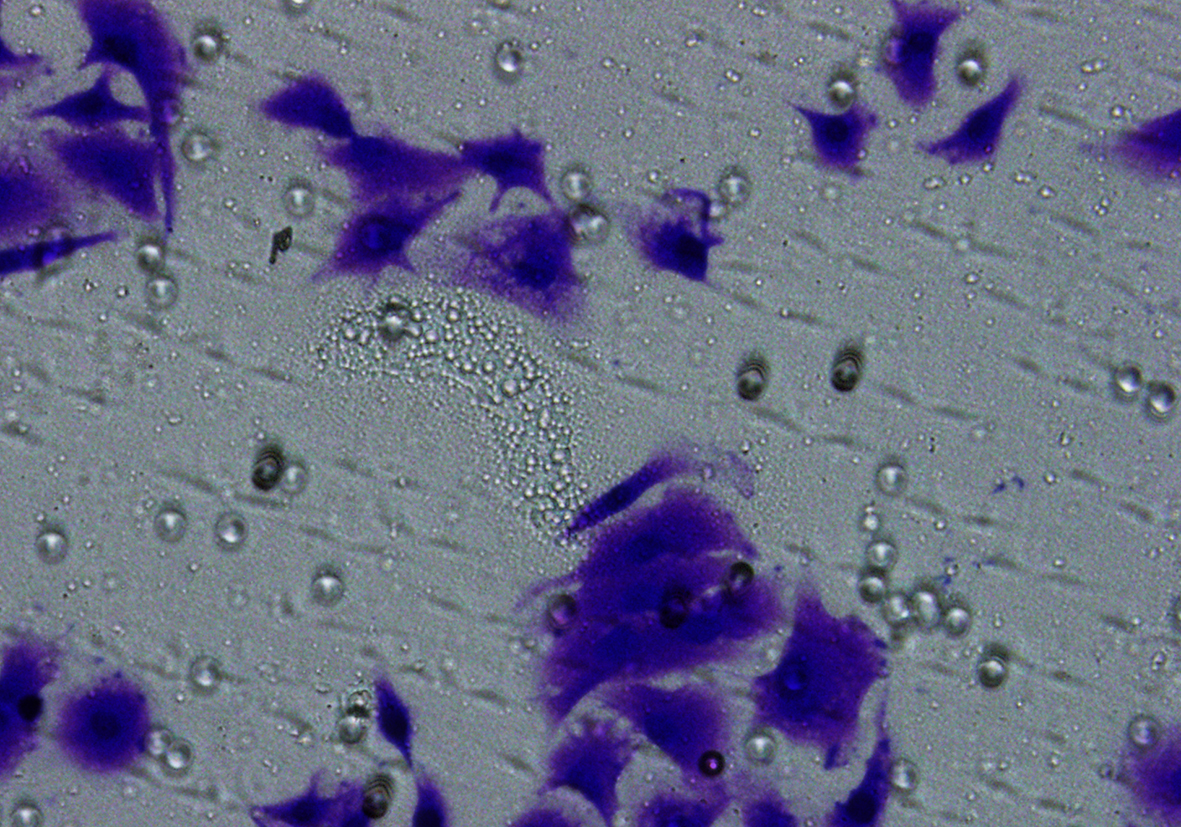

Supplement: Supplemental Information 5 [file peerj-12-17082-s005.zip › raw data/cell numbers/SGC-7901-SI-M/SGC7901-SI-M (2).jpg]

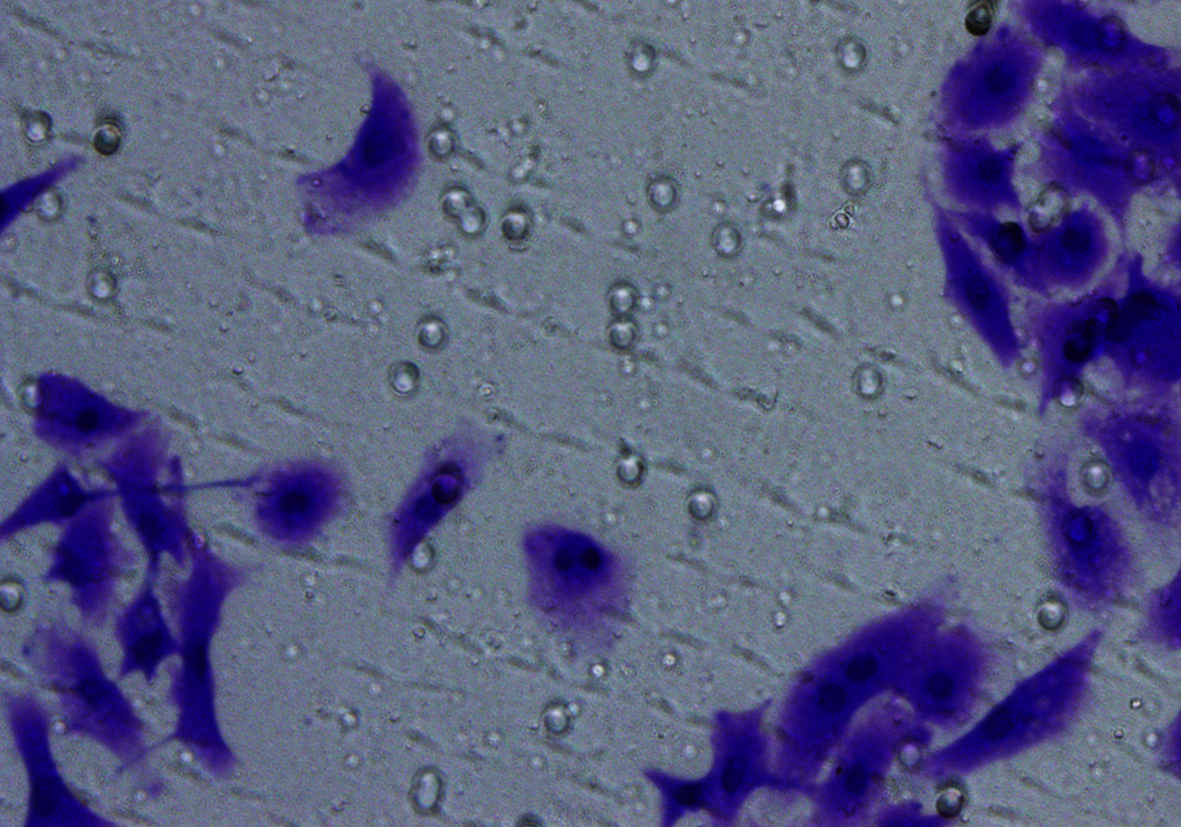

Supplement: Supplemental Information 5 [file peerj-12-17082-s005.zip › raw data/cell numbers/SGC-7901-SI-M/SGC7901-SI-M (3).jpg]

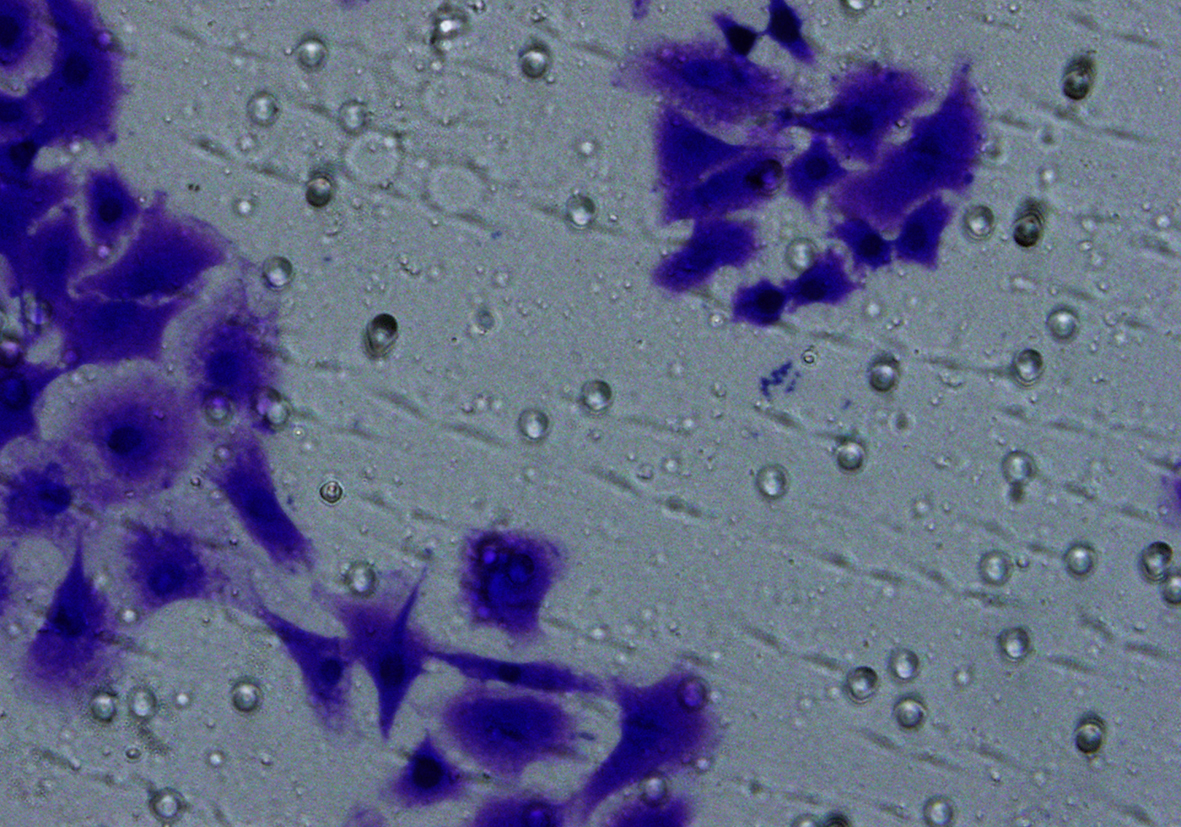

Supplement: Supplemental Information 5 [file peerj-12-17082-s005.zip › raw data/cell numbers/SGC-7901-SI-M/SGC7901-SI-M (4).jpg]

**A**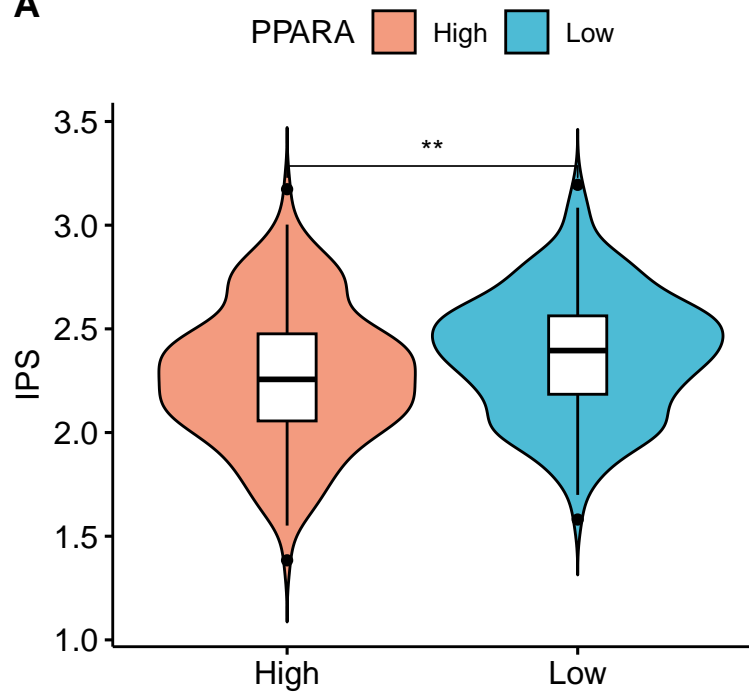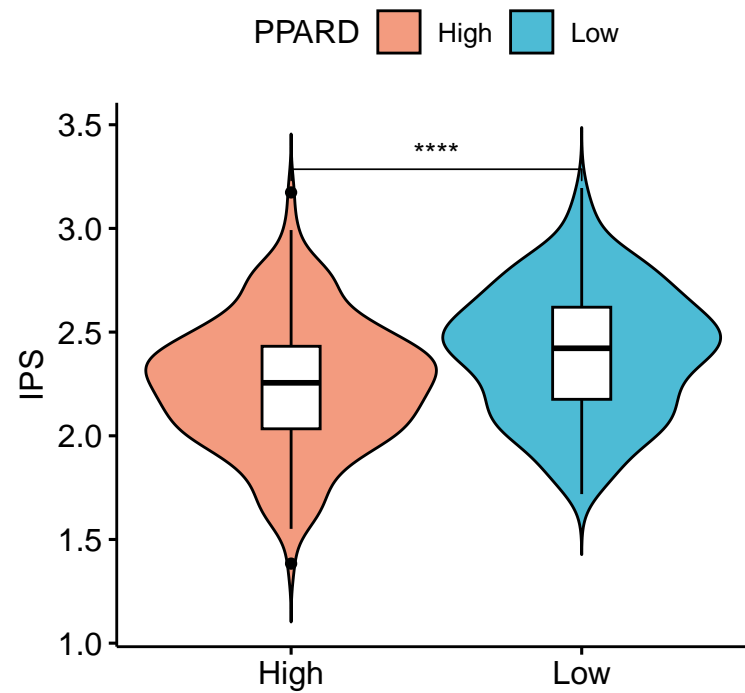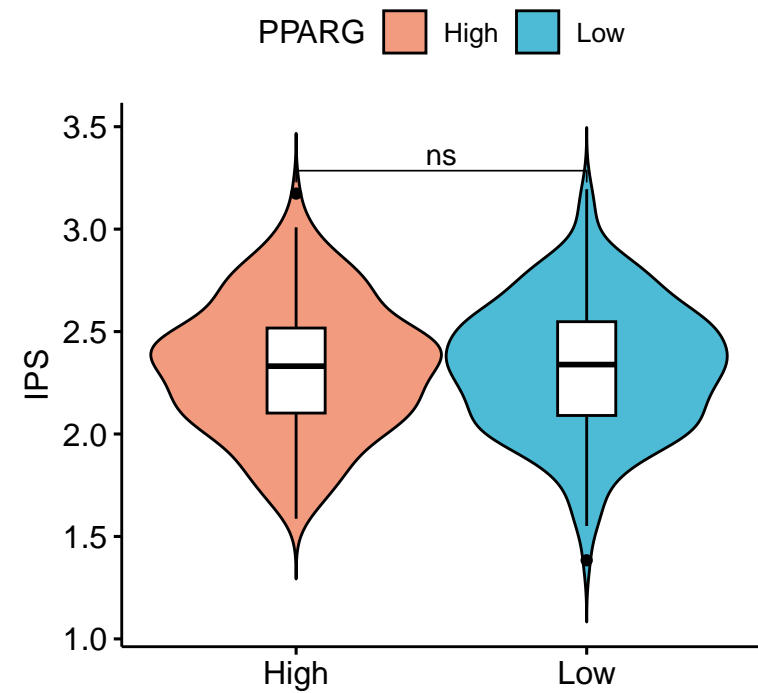**B**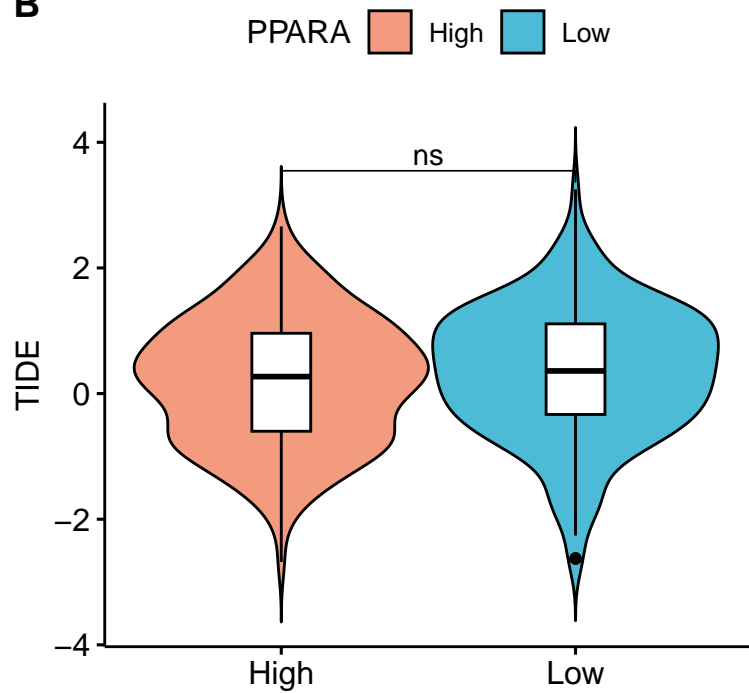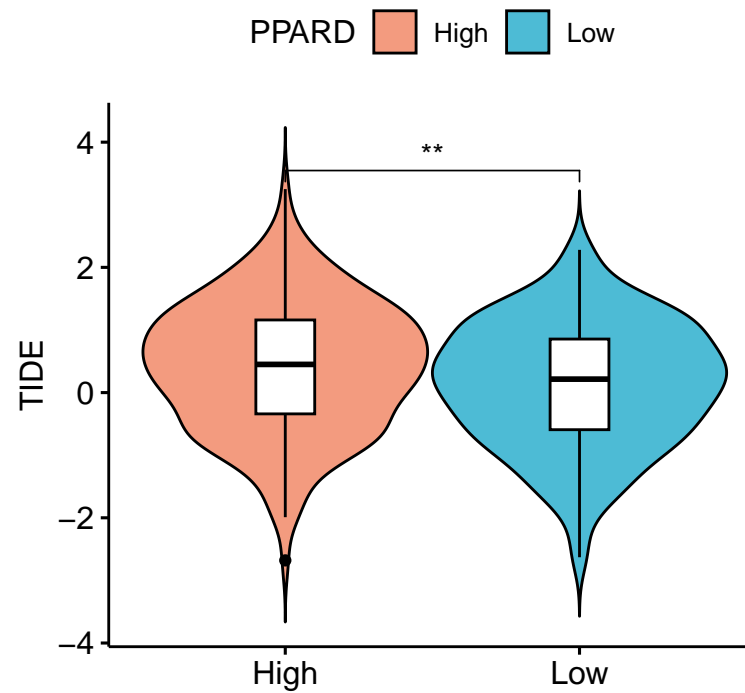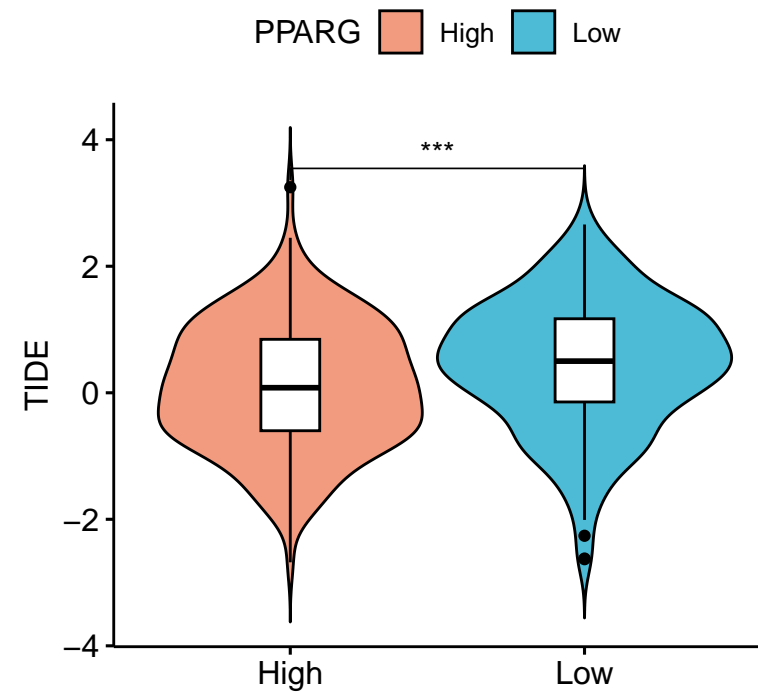

Supplement: Supplemental Information 6 — (A) The immune cell proportion score (IPS) analysis between the high and low-PPAR genes expression groups. (B) The Tumor Immune Dysfunction and Exclusion (TIDE) score analysis between the high and low-PPAR genes expression groups. [file peerj-12-17082-s006.pdf]
