# Supplementary material for: Comprehensive analysis of peroxisome proliferator-activated receptors to predict the drug resistance, immune microenvironment, and prognosis in stomach adenocarcinomas
Source: PeerJ. 2024 Mar 22;12:e17082. doi: 10.7717/peerj.17082 (PMC10962337; doi:10.7717/peerj.17082)

## Slide 1
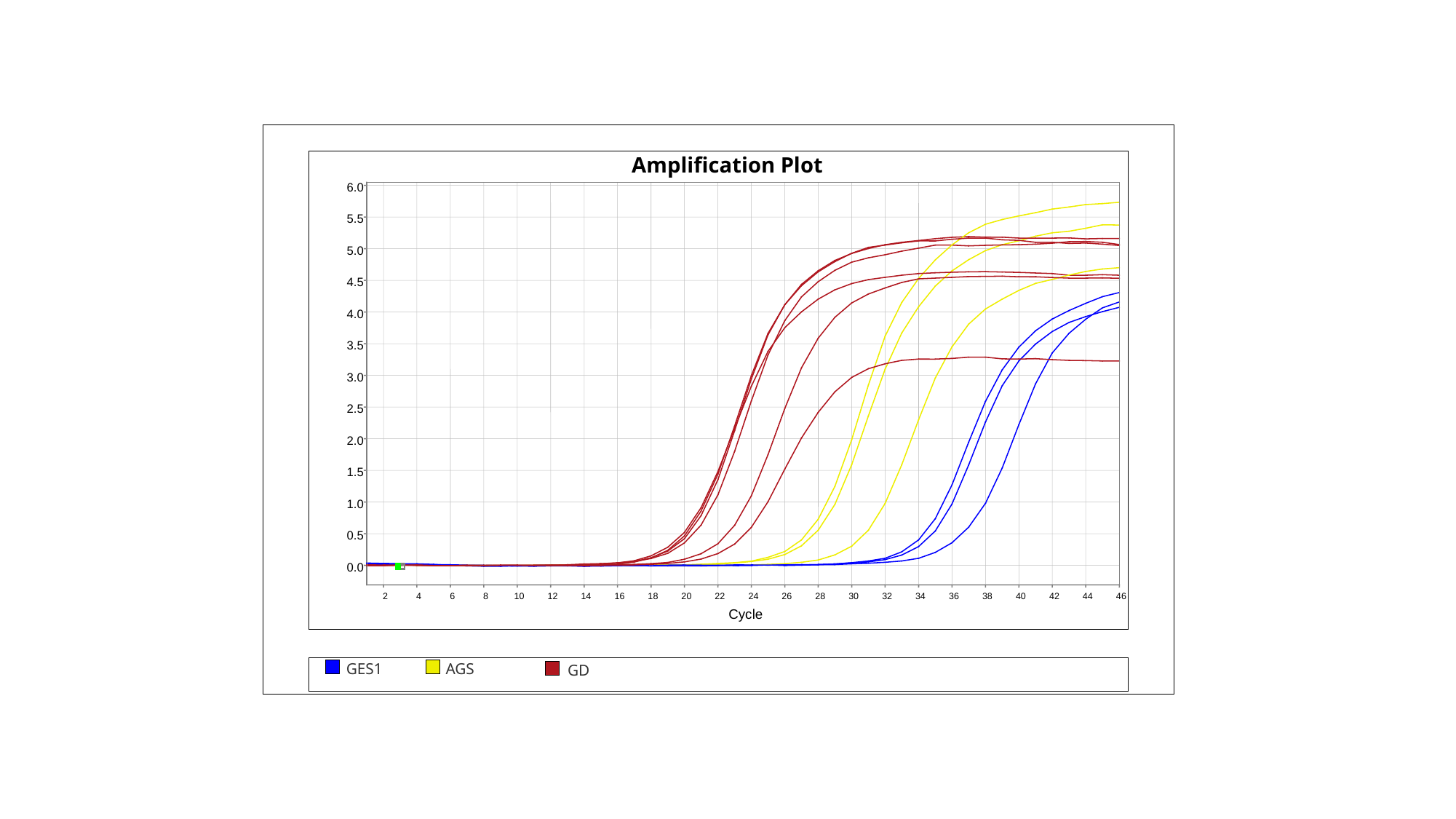

Amplification Plot
6.0
5.5
5.0
4.5
4.0
3.5
3.0
2.5
2.0
1.5
1.0
0.5
0.0
2
4
6
8
10
12
14
16
18
20
22
24
26
28
30
32
34
36
38
40
42
44
46
Cycle
GES1
AGS
GD

## Slide 2
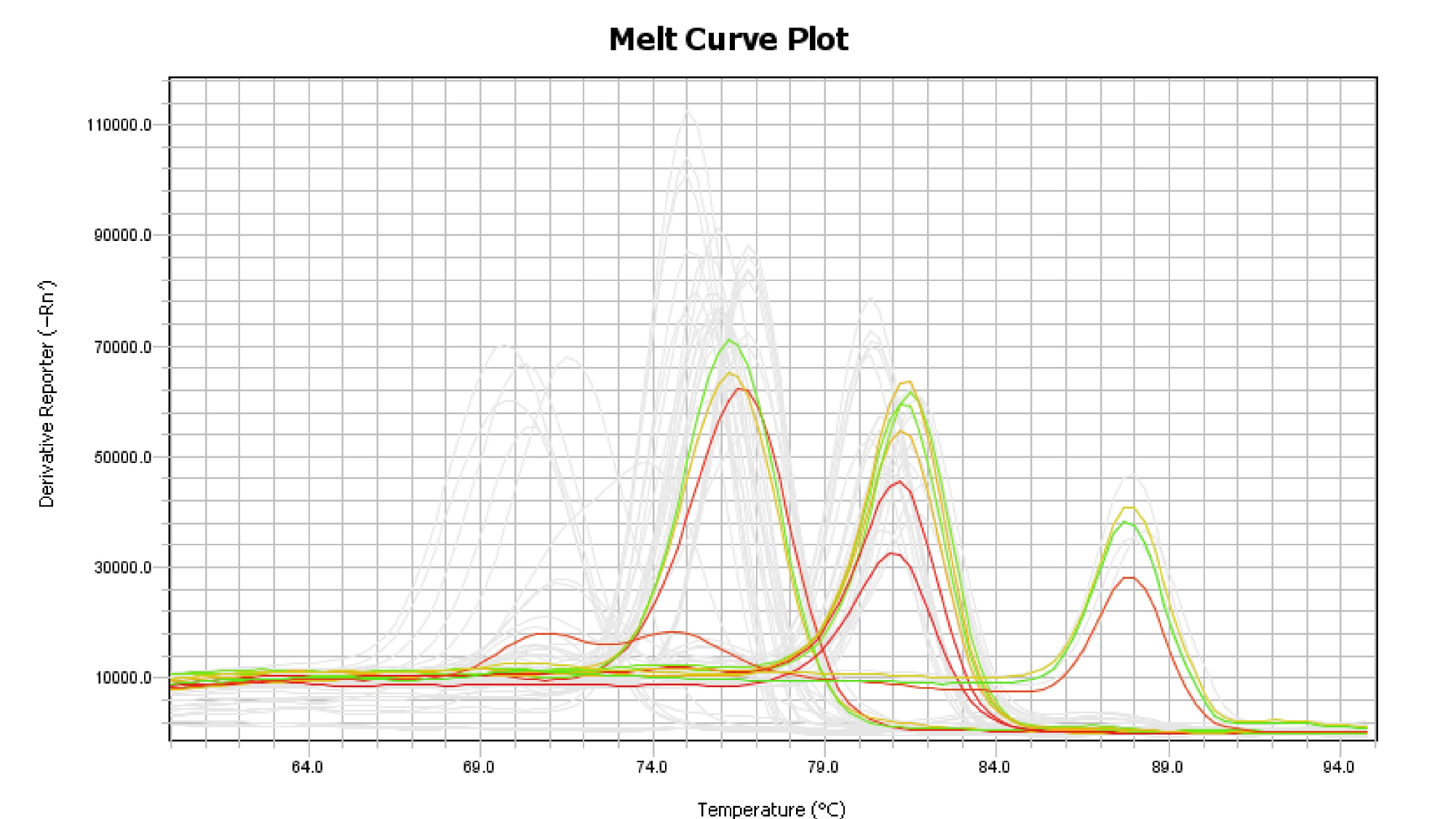

## Slide 3
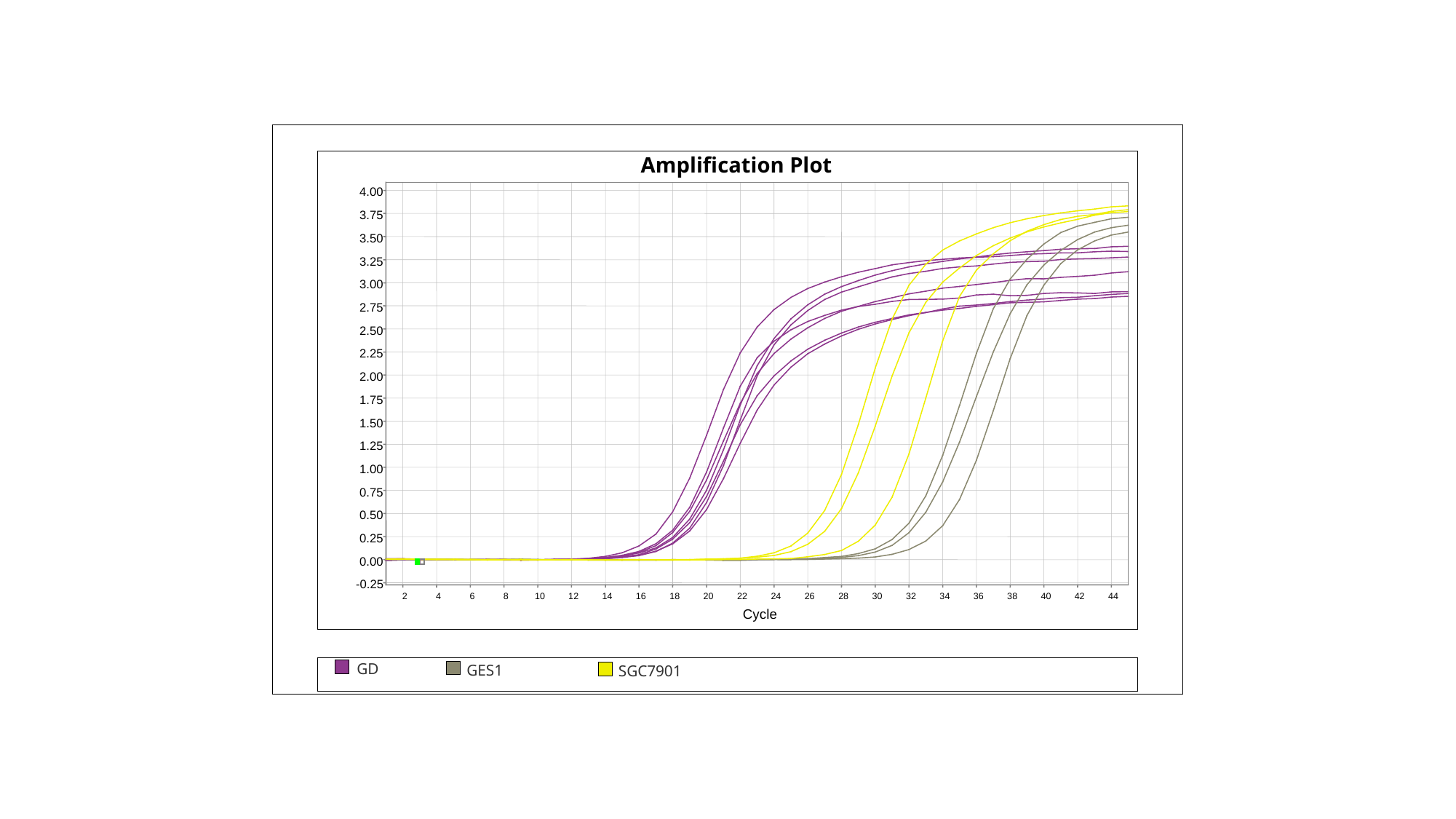

Amplification Plot
4.00
3.75
3.50
3.25
3.00
2.75
2.50
2.25
2.00
1.75
1.50
1.25
1.00
0.75
0.50
0.25
0.00
-0.25
2
4
6
8
10
12
14
16
18
20
22
24
26
28
30
32
34
36
38
40
42
44
Cycle
GD
GES1
SGC7901

## Slide 4
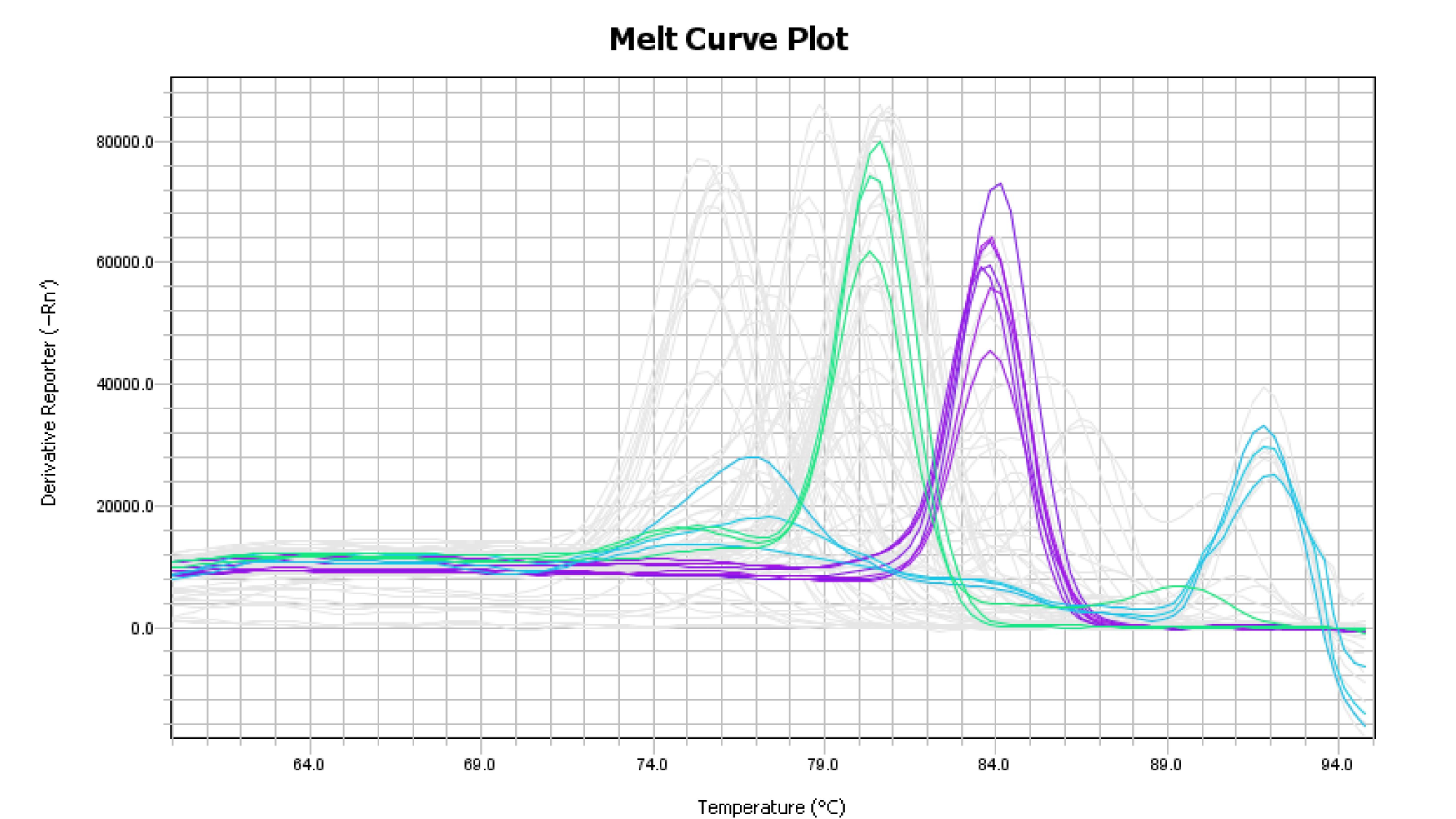

Supplement: Supplemental Information 5 [file peerj-12-17082-s005.zip › raw data/PCR.pptx]
